# Harnessing the anti-inflammatory, skin-protective, and antioxidant potential of *Epilobium dodonaei* extracts using *in vitro* and *in silico* approaches

**DOI:** 10.1039/d6ra01632d

**Published:** 2026-04-08

**Authors:** Inci Kurt-Celep, Gaia Cusumano, Giancarlo Angeles Flores, Gregorio Peron, Ismail Senkardes, Paola Angelini, Carla Emiliani, Abdullahi Ibrahim Uba, Gokhan Zengin

**Affiliations:** a Department of Pharmaceutical Biotechnology, Faculty of Pharmacy, İstanbul Okan University Tuzla İstanbul 34940 Turkey; b Department of Chemistry, Biology and Biotechnology, University of Perugia Via del Giochetto 06123 Perugia Italy carla.emiliani@unipg.it paola.angelini@unipg.it gaia.cusumano@dottorandi.unipg.it; c Botanic Garden “Giardino dei Semplici”, Department of Pharmacy, “Gabriele d’Annunzio” University 66100 Chieti Italy giancarlo.angelesflores@unich.it; d Department of Molecular and Translational Medicine, University of Brescia Brescia Italy gregorio.peron@unibs.it; e Department of Pharmaceutical Botany, Pharmacy Faculty, Marmara University Istanbul Turkey; f Department of Biostatistics and Medical Informatics, Faculty of Medicine, İstinye University 34396 İstanbul Turkey abdullahi.uba@istinye.edu.tr; g Department of Biology, Science Faculty, Selcuk University Konya Turkey gokhanzengin@selcuk.edu.tr

## Abstract

The genus*Epilobium* is widely used for several purposes, including wound healing and the treatment of prostate cancer. In this context, we investigated one *Epilobium* member, namely, *E. dodonaei* extracts for antioxidant, enzyme inhibition, inflammation, oxidative stress and matrix degradation in lipopolysaccharide (LPS)-induced human dermal fibroblasts (LPS + HDF). The extracts were also characterized by ultra-performance liquid chromatography quadrupole time of flight mass spectrometry (UPLC-QToF). In general, the methanol extract contained the highest phenolic (145.38 mg gallic acid equivalent (GAE) g^−1^) and flavonoid (34.82 mg rutin equivalent (RE) g^−1^) content and exhibited the best antioxidant properties (2,2-diphenyl-1-picrylhydrazyl (DPPH):494.40 mg trolox equivalent (TE) g^−1^; cupric reducing antioxidant power (CUPRAC): 881.03 mg TE g; phosphomolybdenum assay: 3.48 mmol TE g^−1^) compared to the ethyl acetate and water extracts. The methanol and ethyl acetate extracts exhibited more substantial enzyme-inhibitory effects than the water extracts. Oenothein B, pedunculagin, galloyl glucose and ellagic acid were the predominant compounds based on the chemical profile. The Water-Soluble Tetrazolium 1 (WST-1) assay confirmed cell viability; protein synthesis of nuclear factor-kappa B (NF-κB) and activator protein-1 (AP-1) transcription factors, and of interleukin-6 (IL-6), interleukin-11 (IL-11), and interferon gamma (IFN-γ), was determined by western blot. Matrix metalloproteinase-2 (MMP-2) and matrix metalloproteinase-9 (MMP-9) activities, which are involved in extracellular matrix (ECM) homeostasis, were measured by gelatin zymography, and gene expression levels were measured. Cellular oxidative stress was assessed using the diacetyldichlorofluorescein (DCFDA) assay. The results suggest that *E. dodonaei* extracts may be considered potential phytotherapeutic agents to promote dermal healing with their multi-targeted anti-inflammatory and antioxidant properties.

## Abstract 2

*Epilobium dodonaei* can be considered as a potential raw material for health-promoting applications.

## Graphical abstract

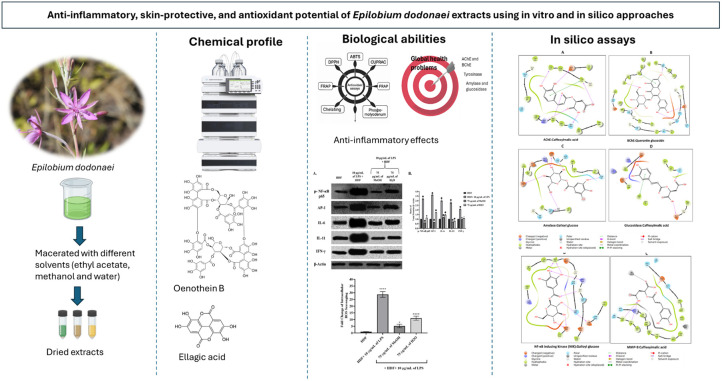

## 1 Introduction

Drug resistance represents an important challenge in modern medicine, as pathogens and cancer cells are increasingly developing resistance to conventional treatments.^1,2^ Natural products, especially those derived from plants, have played a fundamental role in both ancient and modern medicine. Medicinal plants, used for millennia to treat numerous disorders, are an invaluable resource for the discovery and development of new drugs. In modern medicine, these products are particularly important in combating drug resistance, offering natural and often more effective alternatives compared to synthetic compounds. Plants produce a wide range of secondary metabolites with medicinal properties, including alkaloids, flavonoids, and terpenoids, which have been studied and used to develop important drugs.^3^ Some examples of plant-derived compounds include morphine from *Papaver somniferum*, used as a powerful analgesic, paclitaxel from *Taxus brevifolia*, employed in cancer treatment, and artemisinin from *Artemisia annua*, effective against drug-resistant malaria. These examples demonstrate the ongoing value of plants as a source of new therapeutic agents.^4^

Chronic inflammation and oxidative stress are pathological conditions associated with many skin problems, including epidermal barrier disruption, photo-aging, and chronic wound-healing defects.^5^ These conditions are characterized by an increase in reactive oxygen species (ROS), proinflammatory cytokines and extracellular matrix (ECM) degradation enzymes.^6^ Excess secretion of enzymes that break down the ECM, such as collagenase, elastase, and hyaluronidase, enhances the breakdown of dermal collagen fibers, elastic fibers, and hyaluronic acid, leading to the disturbance of skin integrity, wrinkle formation, and the persistence of inflammatory processes.^7^ Therefore, multi-component herbal products that both reduce oxidative stress and target inflammatory signaling pathways and ECM-degrading enzymes stand out as promising phytotherapeutic candidates with the potential to influence dermal inflammation and aging related to inflammation.^8^

The genus *Epilobium*, belonging to the Onagraceae family, comprises roughly 200 distinct species distributed worldwide, showcasing remarkable diversity and adaptability to diverse environmental conditions.^9^ This genus is recognized as a complex group due to frequent interspecific hybridization and significant morphological overlaps between species.^10,11^ Some *Epilobium* species have been widely used in traditional medicine due to their beneficial attributes towards human health.^12^ The use of this plant is, for example, a common practice in Russia, where some parts are used or fermented into an infusion to treat sleep disorders, gastric ulcers, and gastritis.^9^ Native Americans used the herb and root of some *Epilobium* species externally to treat skin infections and rectal bleeding due to their astringent properties.^13^ In Turkey, the genus *Epilobium* is represented by 21 species.^14^ Among them *E*. *angustifolium* and *E*. *hirsutum* are the most common and they are known as yaki in Turkey, are used to treat prostate diseases. Furthermore, *E. hirsutum* is also used to stop bleeding and treat gastrointestinal problems, menstrual disorders, and sleep disorders.^12^ Extracts derived from *E. angustifolium* are widely used for their beneficial effects on benign prostatic hyperplasia (BPH) symptoms, though human clinical trials remain limited. Species of the genus *Epilobium* have attracted increased interest in recent years due to their phytochemical composition. Among *Epilobium* species, for example, the phytochemical composition of *E*. *dodonaei* L. includes various bioactive compounds with potential medicinal properties. The leaves are rich in flavonoids like kaempferol and quercetin, which exhibit antioxidant, anti-inflammatory, and anticancer activities.^15–21^ The herb contains unique flavonoids and tannins, including oenothein B, which is known for its anti-inflammatory and antioxidant effects.^22–24^ The seeds are a source of essential fatty acids and tocopherols, contributing to cardiovascular health and providing antioxidant benefits.^22,24–26^ This diverse phytochemical profile suggests that different parts of *E*. *dodonaei* could be used for various therapeutic purposes, such as reducing inflammation, protecting against oxidative stress, and potentially preventing cancer. Since *E. dodonaei* is a less-studied species and there are no recent studies on its medicinal properties in literature, this species warrants further investigation and analysis.

This work aims to test extracts prepared from the aerial parts of the plant in water (H_2_O), methanolic (MeOH), and ethyl acetate (EA) solutions. The chemical characterization of the tested extracts was performed using UPLC coupled to high-resolution MS (QToF). The various extracts were tested for their ability to scavenge free radicals, chelate metal ions, and reduce phosphomolybdenum. Additionally, the ability of these extracts to inhibit acetylcholinesterase, tyrosinase, α-amylase and α-amylase enzymes was also evaluated. In addition, investigation of the effects of the extracts on LPS-induced inflammatory response and ECM degradation parameters (cytokine profile, MMP/ECM enzyme balance, *etc.*) in human dermal fibroblasts is important both for revealing the pharmacological potential of this understudied species and for evaluating a new herbal candidate in the phyto-therapeutic management of skin disorders associated with chronic inflammation.

## 2 Materials and methods

### 2.1 Plant collection

Plant specimens were collected in 2023 from the Ilgaz Mountain (Kastamonu, Turkey) at an elevation of 1280 m. Dr Ismail Senkardes carried out the formal botanical classification of the material. A voucher specimen, under the code MARE-22452, was placed in the Faculty of Pharmacy at Marmara University. After collection, the above-ground parts were separated immediately and air-dried at room temperature in a shaded, well-ventilated area. After complete drying, the plant material was milled to a fine powder. To ensure chemical integrity and prevent deterioration, the powdered samples were sealed in opaque containers and stored under stable conditions.

### 2.2 Plant extract preparation

Three solvent systems were used to obtain bioactive constituents: ethyl acetate, methanol, and distilled water. For each extraction, 10 g of the prepared plant material was mixed with 200 mL of the corresponding solvent. The methodology was adapted according to the properties of the solvent. Extractions using organic solvents were performed *via* maceration for 24 hours under ambient conditions. In contrast, the water-based extraction utilised a 15-minutes infusion with heated water. Following extraction, the products obtained were concentrated using distinct techniques. The aqueous extract was stabilised by lyophilisation, while the organic solvent fractions were recovered by evaporating the solvents under reduced pressure using a rotary evaporator. Extraction yields (%) are given in Table 1.

**Table 1:** Extraction yields (%) and total bioactive components in the tested extracts^a^

Extracts	Extraction yields (%)	Total phenolic content (mg GAE g^−1^)	Total flavonoid content (mg RE g^−1^)
EA	7.17	35.64 ± 0.80^c^	29.26 ± 0.76^b^
MeOH	19.84	145.38 ± 4.77^a^	34.82 ± 0.31^a^
H_2_O	16.43	141.28 ± 2.29^ab^	20.51 ± 0.26^c^

a Values expressed are means ± S.D. of three parallel measurements. EA: ethyl acetate extract, MeOH: methanol extract, H_2_O: water extract. GAE: gallic acid equivalent; RE: rutin equivalent. Different letters indicate significant differences among the tested extracts (*p* < 0.05).

### 2.3 Assay for total phenolic and flavonoid contents

Total phenolic and flavonoid contents of the extracts were determined using established colorimetric assays (Folin–Ciocalteu assay for total phenolic content; AlCl_3_ assay for total flavonoid content), following the referenced procedure. Calibration curves were constructed using gallic acid (mg gallic acid equivalents (GAE) g^−1^) and rutin (mg rutin equivalents (RE) g^−1^) as standards.^27^

### 2.4 Phytochemical characterization of extracts

Approximately 50 mg of each dried extract was accurately weighed and dissolved in 1 mL of HPLC-grade methanol. The mixtures were subjected to sonication in an ultrasonic bath for 10 minutes to ensure complete dissolution and compound extraction. Following sonication, the samples were centrifuged at 13 300 rpm for 10 minutes to pellet any suspended particulate matter. The resulting supernatants were carefully collected and filtered through 0.22 µm membrane filters prior to chromatographic analysis. Chemical profiling was conducted using ultra-performance liquid chromatography coupled with high-resolution mass spectrometry (UPLC-HRMS). Analyses were performed on a Waters Acquity UPLC system interfaced with a Xevo G2 Q-ToF mass spectrometer equipped with an electrospray ionization (ESI) source. All chromatographic details are given in the SI materials.

### 2.5 Assays for *in vitro* antioxidant capacity

Antioxidant capacity was assessed using a panel of *in vitro* assays, following a previously described protocol.^28^ Reducing power was evaluated using FRAP (ferric reducing antioxidant power) and CUPRAC (cupric reducing antioxidant capacity), while radical scavenging was measured using DPPH (2,2-diphenyl-1-picrylhydrazyl) and ABTS (2,2′-azino-bis(3-ethylbenzothiazoline-6-sulfonic acid)) assays. Results from these four assays were quantified and standardized to Trolox and reported as mg Trolox equivalents (TE) per g of dried extract (mg TE g^−1^). Trolox and expressed as milligrams of Trolox equivalent per gram of dried extract (mg TE g^−1^). Total antioxidant capacity was additionally determined by the phosphomolybdenum method (PBD assay) and expressed as mmol Trolox equivalents per g (mmol TE g^−1^). Finally, metal-chelating activity was evaluated using a chelation assay and reported as mg EDTA equivalents (EDTAE) per g of extract (mg EDTAE g^−1^).

### 2.6 Inhibitory effects against some key enzymes

The enzyme inhibitory potential of the extracts was evaluated against five key targets; acetylcholinesterase (AChE), butyrylcholinesterase (BChE), tyrosinase, α-amylase and α-glucosidase; using established colorimetric procedures.^28^ To standardise the results, inhibition was quantified using established reference compounds. Cholinesterase inhibitory activity was expressed as mg galanthamine equivalents (GALAE) per g of extract (mg GALAE g^−1^). α-Amylase and α-glucosidase inhibitory activities were expressed as mg acarbose equivalents (ACAE) per g (mg ACAE g^−1^), while tyrosinase inhibition was reported as mg kojic acid equivalents (KAE) per g (mg KAE g^−1^).

### 2.7 Skin enzyme inhibition tests (collagenase, elastase, hyaluronidase)

Standard colorimetric methods were used to assess the inhibitory activity of *E*. *dodonaei* extracts in water (H_2_O), methanol (MeOH), and ethyl acetate (EA) against extracellular enzyme targets. The final extract concentration was adjusted to 1 mg mL^−1^ in all experiments. For the collagenase inhibition assay, reactions were prepared in 50 mM Tris–HCl buffer (pH 7.4) using Clostridium histolyticum collagenase and FALGPA as substrate, and the absorbance decrease at 340 nm was recorded after incubation. The elastase inhibition assay was performed in 200 mM Tris–HCl buffer (pH 8.0) with porcine pancreatic elastase and *N*-succinyl-Ala-Ala-Ala-*p*-nitroanilide substrate, measuring the release of *p*-nitroaniline at 410 nm. For hyaluronidase inhibition, bovine testicular hyaluronidase was incubated in 0.1 M acetate buffer (pH 4.5) containing sodium hyaluronate as substrate, and the residual turbidity was quantified at 600 nm.^29,30^

All assays included enzyme controls and blanks; inhibition percentages were calculated relative to the uninhibited enzyme activity. Epigallocatechin gallate (EGCG, 250 µg mL^−1^) served as the positive control for anti-collagenase and anti-elastase assays, while tannic acid (250 µg mL^−1^) was used for the anti-hyaluronidase test. Each assay was conducted in triplicate, and results were expressed as mean ± SD.^29,30^ The enzymes employed in the skin enzyme (collagenase, elastase, hyaluronidase) inhibition assays were purchased from Sigma-Aldrich (St. Louis, MO, USA). Specifically, collagenase derived from Clostridium histolyticum (EC 3.4.24.3), porcine pancreatic elastase (EC 3.4.21.36), and hyaluronidase obtained from bovine testes (EC 3.2.1.35) were used. The enzyme preparations were applied at appropriate activity or concentration ranges as required for each assay.

### 2.8 Cell culture

Human dermal fibroblast (HDF) cells were cultured in Dulbecco's Modified Eagle Medium (DMEM) supplemented with 10% fetal bovine serum (FBS), 1% l-glutamine, and 1% penicillin-streptomycin (100 U per mL penicillin and 100 µg mL^−1^ streptomycin) under standard conditions (37 °C, 5% CO_2_). Upon reaching approximately 85% confluency, the cells were detached using 0.25% trypsin–EDTA and passaged for subsequent experiments. Cryopreserved aliquots of early-passage HDFs were maintained in liquid nitrogen to ensure cellular consistency across experiments.^31^ To simulate an inflammatory microenvironment, cells were exposed to lipopolysaccharide (LPS, from *E. coli* O111:B4) at a final concentration of 10 µg mL^−1^ for 48 h, which has been shown to induce strong pro-inflammatory responses (IL-6, TNF-α, and IL-17 upregulation).^31^ After LPS treatment, cells were rinsed with PBS and incubated with *E*. *dodonaei* extracts (methanolic or aqueous, 25–100 µg mL^−1^) for 48 h to evaluate the modulation of inflammation-associated pathways. Non-treated HDFs (neither exposed to LPS nor extracts) served as the negative control, representing basal cellular physiology in all *in vitro* experiments.

The methanolic and aqueous extracts were specifically selected for the cell-based assays due to their previously confirmed high phenolic and flavonoid content, potent antioxidant and ROS-scavenging capacity, and their non-toxic nature in fibroblast models. These extracts also demonstrated significant inhibition of skin-aging/-damage-related enzymes (collagenase, elastase, and hyaluronidase) *in vitro*, indicating a dual mechanism relevant to oxidative and inflammatory modulation. The selection is consistent with previous findings on *E*. *angustifolium* and *E. hirsutum* where methanolic and aqueous extracts exhibited optimal biological activity and cytocompatibility.^6,12^ Also hTERT-immortalized normal human dermal fibroblasts (CRL-4066) were obtained from the American Type Culture Collection (ATCC, Manassas, VA, USA) and have been previously employed in our studies to ensure methodological consistency and reproducibility.

### 2.9 Cell viability assay by WST-1

To determine the non-toxic concentration range of *E*. *dodonaei* extracts for use in HDF cells, a WST-1 assay, which is based on the reduction of tetrazolium salts by mitochondrial dehydrogenases, was performed. Briefly, HDF cells were seeded at a density of 1 × 10^4^ cells per well in 96-well plates and allowed to adhere for 16 hours. Following attachment, methanolic and water extracts of *E. dodonaei* were applied at concentrations ranging from 25 to 100 µg mL^−1^ and incubated for 48 hours. At the end of the treatment period, 10% (v/v) WST-1 reagent was added to each well and incubated for 1 hour. Absorbance was then measured at 570 nm. Cell-viability values were normalized to those of untreated HDF cells and reported as percent viability. A viability level of ≥60% was considered the threshold for “non-toxic,” and the identified non-cytotoxic concentration was used in subsequent *in vitro* experiment.^6,32–34^

### 2.10 Western Blot

Western blot analysis was performed to evaluate the regulatory effects of *E*. *dodonaei* extracts on the LPS-induced inflammatory response. HDF cells were divided into four groups: control, LPS, LPS + MeOH extract (75 µg mL^−1^), and LPS + H_2_O extract (75 µg mL^−1^). After incubation with 10 µg mL^−1^ LPS, the cells were treated with 100 µg mL^−1^ extracts determined to be non-toxic by WST-1 for 48 hours.^31^ Cells were lysed with RIPA buffer, and total protein was measured by the BCA method, and 30 µg of protein from each sample was loaded onto 15% SDS-PAGE gels. Separated proteins were transferred to PVDF membranes, blocked with 5% skim milk, and incubated with antibodies to NF-κB p65, AP-1, IL-6, IL-11, IFN-γ, and β-actin. Bands were visualized with ECL substrate after HRP-conjugated secondary antibodies. Densitometry analyses were performed using ImageJ software, and all proteins were normalized to β-actin to calculate relative expression levels according to the LPS group.^29,31,32^

### 2.11 Gelatine zymography

Gelatin zymography analysis was performed to evaluate the modulation of LPS-induced matrix metalloproteinase activity in HDF cells by *E*. *dodonaei* extracts. Cells were divided into four groups: control, LPS, LPS + MeOH extract (75 µg mL^−1^), and LPS + H_2_O extract (75 µg mL^−1^). Cells were incubated with LPS (10 µg mL^−1^) for 48 hours and then treated with 75 µg mL^−1^ extracts determined to be non-toxic for 48 hours. At the end of the 48th hour after all treatments, culture supernatants were collected and loaded onto zymography gels containing 0.1% gelatine. Following cold electrophoresis, the gel was incubated in developing buffer to reveal enzyme activity. The following day, gels were stained with Coomassie Brilliant Blue, and lytic background bands indicating gelatinase activity were observed. Band intensities corresponding to MMP-2 and MMP-9 were visualized with ChemiDoc and analysed using ImageJ software. Analyses were reported in comparison with LPS groups, thus determining the regulatory effects of the extracts on LPS-induced MMP activity.^29,31,32^

### 2.12 RT-PCR

RT-qPCR analysis was performed to evaluate the effects of *E*. *dodonaei* extracts on LPS-induced MMP-2 and MMP-9 gene expressions. HDF cells were divided into four groups: control, LPS, LPS + MeOH extract (75 µg mL^−1^), and LPS + H_2_O extract (75 µg mL^−1^). Cells were incubated with LPS (10 µg mL^−1^) for 48 hours and then treated with 75 µg mL^−1^ concentrations of the extracts, which were determined to be non-toxic, for 48 hours. Total RNA isolation was performed using peqGOLD TriFast™ (Trizol-based, Peqlab) reagent according to the manufacturer's protocol. The purity and quantity of RNA were assessed spectrophotometrically, and cDNA synthesis from equal amounts of RNA was performed using the Sensiscript Reverse Transcription Kit (QIAGEN). qPCR reactions were conducted using QIAGEN SYBR® Green PCR Master Mix. 18S rRNA was selected as the housekeeping gene. Ct values were obtained from three technical replicates, and gene expression analyses were calculated as relative fold change according to the LPS group.

### 2.13 Cellular antioxidant with DCFDA

DCFDA-based cellular ROS analysis was performed to assess the modulation of LPS-induced oxidative stress in HDF cells by *E*. *dodonaei* extracts. Cells were divided into four groups: control, LPS, LPS + MeOH extract (75 µg mL^−1^), and LPS + H_2_O extract (75 µg mL^−1^). Cells were incubated with LPS (10 µg mL^−1^) for 48 hours and then treated with extracts determined to be non-toxic for 48 hours. At the end of incubation, cells were washed with PBS and loaded with 10 µM DCFDA solution for 30 minutes at 37 °C under dark conditions. The probe, converted to DCFH by esterase activity, was oxidized in the presence of intracellular ROS to form the fluorescent DCF form. After loading, cells were washed again with PBS, and fluorescence intensity was measured at 485/535 nm (Ex/Em) wavelengths in a microplate reader.The obtained fluorescence values were normalized to the control group, and ROS levels were calculated as fold change.^29,31^

### 2.14 Molecular modeling

Three-dimensional structures of the target proteins were retrieved from the Protein Data Bank (PDB; https://www.rcsb.org/).^35^ The selected crystal structures included: human acetylcholinesterase (AChE) in complex with donepezil (PDB ID: 7E3H),^36^ human butyrylcholinesterase (BChE) with inhibitor AI0 (PDB ID: 6EQP),^37^ pancreatic α-amylase bound to acarbose (PDB ID: 1B2Y),^38^ tyrosinase from *Bacillus megaterium* complexed with inhibitor JKB (PDB ID: 6QXD),^39^ NF-κB inducing kinase (NIK) in complex with an inhibitor (PDB ID: 7SZR),^40^ and the catalytic domain of MMP-9 bound to a hydroxamate-based inhibitor (PDB ID: 4WZV).^41^ For glucosidase, a previously developed homology model from our research group was used.^42^

Protein structures were prepared using the ProteinPrepare tool available on the PlayMolecule server (https://www.playmolecule.com/), which calculates the p*K*_a_ values of titratable residues and applies the corresponding protonation states.^43^ Ligand structures were downloaded from the PubChem database (https://pubchem.ncbi.nlm.nih.gov/)^44^ and geometry-optimized using UCSF Chimera.^45^ For docking grid generation, each co-crystallized ligand was used as a reference, and AutoDockTools (ADT) was employed to set the grid box dimensions to 45 × 45 × 45 Å along the *X*, *Y*, and *Z* axes. Hydrogen atoms were merged, and Gasteiger partial charges were assigned to all atoms before docking.

Molecular docking was performed using AutoDock 4.2.6 (https://autodock.scripps.edu/)^46^ with the Lamarckian genetic algorithm for ligand conformational search. Due to their large molecular size, oenothein B, oenothein A, and pedunculagin could not be accommodated in the active sites of the selected enzymes; therefore, these compounds were docked into predicted allosteric pockets using SwissDock with the “attracting cavities” option.^47^ Protein–ligand interactions were visualized and analyzed in Maestro Viewer (Schrödinger, Inc).

### 2.15 Statistical analysis

All experiments conducted within the scope of this study were repeated at least three different time periods independently. Statistical analyses of the obtained data were performed using version 10.1.1 of the GraphPad Prism statistical analysis software. In skin-related enzyme inhibition experiments (collagenase, elastase, and hyaluronidase), each enzyme was compared with its own specific standard, and One-Way ANOVA statistical analysis was applied to evaluate differences between groups. In WST-1 analyses, where cell proliferation and cytotoxicity were evaluated simultaneously, increasing concentrations of plant extracts were applied to cells incubated for 48 hours, and the results were analyzed using the One-Way ANOVA method. Similarly, in gelatin zymography, RT-PCR, western blot, and cellular oxidative stress experiments, where untreated HDF cells were considered as the control group, statistical differences between groups were evaluated using One-Way ANOVA and appropriate multiple comparison tests. The lowest level of statistical significance was accepted as *p* < 0.05. On the other hand, experimental groups containing similar values when compared to untreated-HDF cells but where no statistically significant difference was observed were expressed as ns.

## 3 Results and discussion

### 3.1 Total phenolic and flavonoid content

In this research, the total phenolic and flavonoid contents in the extracts were evaluated through colorimetric assays. As can be seen from Table 1, the highest presence of phenolics was observed in the methanol extract, with a value of 145.38 ± 4.77 mg GAE g^−1^, followed by water (141.28 ± 2.29 mg GAE g^−1^), and ethyl acetate (35.64 ± 0.80 mg GAE g^−1^). Regarding the total flavonoid content, the methanol extract was the richest with a value of 34.82 ± 0.31 mg RE g^−1^, followed by ethyl acetate (29.26 ± 0.76 mg RE g^−1^), and then water (20.51 ± 0.26 mg RE g^−1^).

In the literature, there are no studies regarding the phenolic and flavonoid composition of *E. dodonaei*. However, the results of total phenolic content are similar with our previous study from 2021,^12^ in which we analyzed the phenolic composition of the aerial part extract of *E. hirsutum*. In that case, the highest amount of phenolics was also found in the extracts prepared in methanol (254.55 mg GAE g^−1^), while the lowest was in the extracts prepared in ethyl acetate (43.52 mg GAE g^−1^). Also, for the total flavonoid content in our previous study we found that the MeOH extract was the richest (87.66 mg RE g^−1^). The variations in total bioactive compounds in these extracts can be explained by the solvent polarity. It has already been reported in the literature that total phenolic content can be influenced by solvent polarity,^48,49^ and similarly, total flavonoid content can be influenced not only by the plant species and the plant part used but also by the type of solvent and the polarity index.^50^ Our results agree with some studies that have found methanol to be more effective than water in extracting phenolic compounds and flavonoids.^51,52^ This could be reflected in a greater antioxidant capacity, as highlighted in our study. According to some reports, other analytical techniques are needed to confirm Folin-Ciocalteu results, because this test does not reflect the true content of phenols.^53^ For these reasons, the different extracts were analyzed using UPLC coupled to high-resolution MS (QToF).

### 3.2 Phytochemical characterization of extracts using UPLC-QToF

**Fig. 1:**
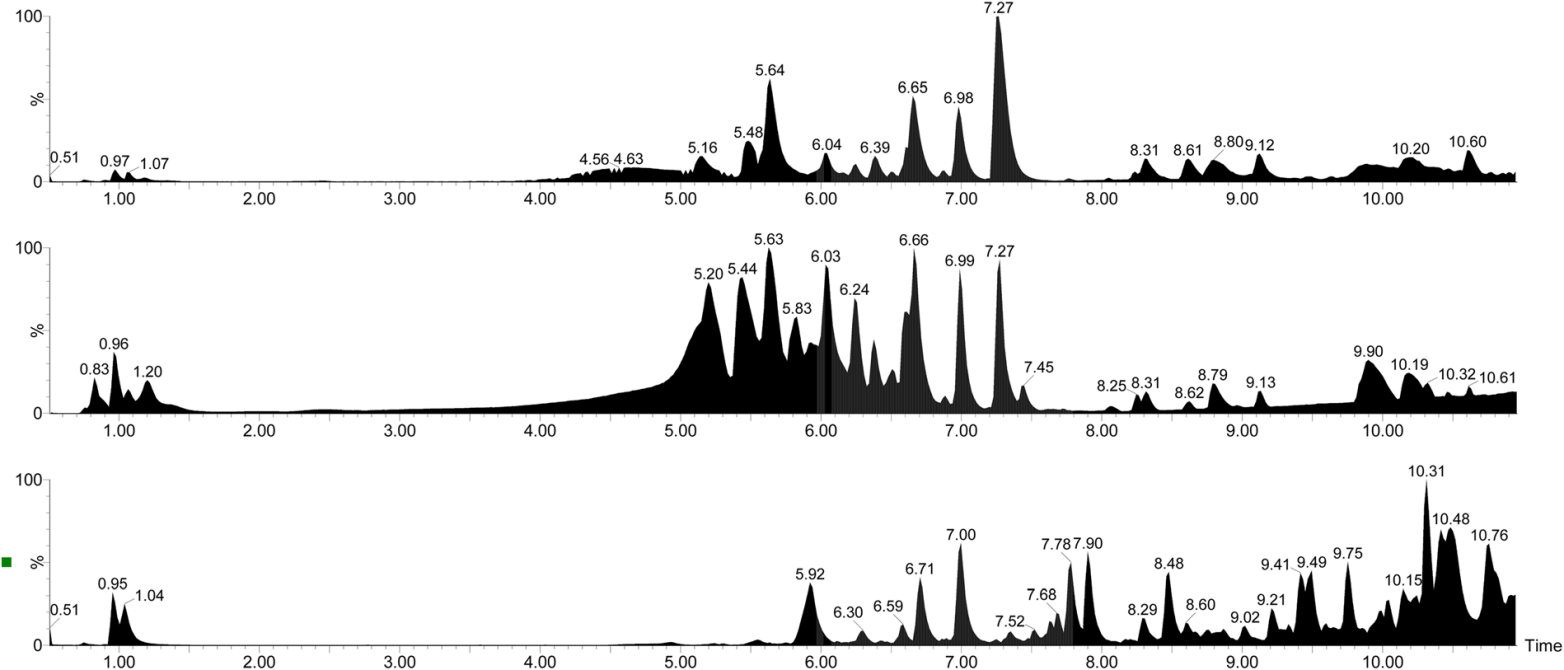
Exemplificative chromatograms of EtAc (upper panel), MeOH (medium panel), and aqueous (lower panel) extracts of *E. dodonaei*. Analyses were performed by UPLC-QToF MS in negative ion mode. Chromatograms were magnified in the 0–11 min RT region to highlight the peaks of identified metabolites.

Extracts were analyzed using UPLC coupled to high-resolution MS (QToF). Overall, the analysis revealed several metabolites in the extracts, among which 46 were identified by means of MS and MS^e^ data and by comparison with the published literature.^6,8,34^ Results are shown in Table 2. Representative chromatograms are reported in Fig. 1. The amount of identified secondary metabolites in the extracts was significantly different, with EtAc and aqueous extracts presenting the highest and lowest amounts, respectively (235 mg g^−1^ and 33 mg g^−1^). This result was expected, since higher yields of extraction of plant secondary metabolites with alcohols and moderately apolar solvents such as EA have been widely reported in the literature.^54^ This behavior is consistent with previous phytochemical investigations on *Epilobium* spp., in which methanol, ethanol, and intermediate-polarity solvents were shown to maximize the extraction of polyphenols, particularly ellagitannins and flavonoids, due to their intermediate polarity and structural diversity.^9,55^

**Table 2:** Secondary metabolites identified in ethyl acetate (EA), methanol (MeOH), and water (H_2_O) extracts of *E. dodonaei* by using UPLC-QToF in negative ionization mode. Quantitative results are reported as mg g^−1^ of extract. Data are shown as mean ± S.D. of *n* = 3 measurements^a^

RT (min)	*m*/*z*	Fragments	Molecular formula of parent compound	Tentative identification (adduct ion type)	EA	MeOH	H_2_O
**Gallic acid derivatives**							
1.38	169.0127		C_7_H_6_O_5_	Gallic acid	2.83 ± 0.04	6.03 ± 0.10	0.01 ± 0.01
2.34	331.0657	169.0127	C_13_H_16_O_10_	Galloyl glucose	1.15 ± 0.03	4.26 ± 0.05	0.04 ± 0.04
4.21	1567.1459		C_68_H_48_O_44_	Oenothein B	3.09 ± 0.16	20.98 ± 0.10	0.01 ± 0.00
4.23	633.0736		C_27_H_22_O_18_	Gemin D	0.18 ± 0.01	0.61 ± 0.02	ND
4.24	1175.1049		C_102_H_72_O_66_	Oenothein A ([M–2H]^2−^)	0.46 ± 0.03	1.40 ± 0.02	ND
4.58	633.0743	169.0127	C_27_H_22_O_18_	Galloyl-HHDP-glucoside	0.16 ± 0.08	1.68 ± 0.05	0.10 ± 0.05
5.17	783.0684		C_34_H_24_O_22_	Pedunculagin	21.4 ± 0.00	8.58 ± 0.01	0.14 ± 0.00
5.35	392.0371		C_34_H_26_O_22_	Sanguiin H1 ([M–2H]^2−^)	1.70 ± 0.03	1.13 ± 0.01	0.01 ± 0.00
5.77	483.0775		C_20_H_20_O_14_	Galloyl glucose	5.91 ± 0.05	12.57 ± 0.02	0.05 ± 0.05
5.85	300.9984		C_14_H_6_O_8_	Ellagic acid	12.60 ± 0.01	11.06 ± 0.01	0.49 ± 0.01
5.94	183.029		C_8_H_8_O_5_	Methyl gallate	0.04 ± 0.03	17.29 ± 0.07	0.02 ± 0.02
5.97	615.0626		C_27_H_22_O_18_	Sanguiin H4 ([M–H_2_O–H]^−^)	1.21 ± 0.02	0.63 ± 0.01	0.01 ± 0.00
6.18	325.056		C_14_H_14_O_9_	Galloylshikimic acid	3.30 ± 0.08	3.33 ± 0.05	1.19 ± 0.03
6.20	433.0407		C_19_H_14_O_12_	Ellagic acid arabinoside	3.34 ± 0.00	2.78 ± 0.00	0.07 ± 0.01
6.30	433.0407		C_19_H_14_O_12_	Ellagic acid xylopiranoside	4.31 ± 0.00	3.30 ± 0.01	0.39 ± 0.01
6.33	491.1765		C_21_H_32_O_1_	Antiarol rutinoside	1.60 ± 0.06	4.27 ± 0.07	0.03 ± 0.03
6.85	939.1118		C_41_H_32_O_26_	Penta-*O*-galloyl-alpha-d-glucopyranose	7.48 ± 0.12	12.19 ± 0.09	ND
6.90	537.1244		C_24_H_26_O_14_	O-Sinapoyl-6′-*O*-galloyl-beta-d-glucose	2.14 ± 0.10	3.86 ± 0.04	0.02 ± 0.03
				**Total identified**	**72.90** ± **0.20**	**115.93** ± **0.06**	**2.58** ± **0.06**

**Phenolic acid**							
5.69	285.061		C_12_H_14_O_8_	Uralenneoside	0.60 ± 0.05	1.35 ± 0.04	0.05 ± 0.01
5.88	247.0243		C_12_H_8_O_6_	Brevifolin	0.30 ± 0.08	1.93 ± 0.07	ND
5.89	291.0135		C_13_H_8_O_8_	Brevifolincarboxylic acid	0.56 ± 0.08	3.98 ± 0.04	0.04 ± 0.01
5.99	163.039		C_9_H_8_O_3_	Coumaric acid	1.46 ± 0.12	3.35 ± 0.04	0.22 ± 0.03
6.00	295.0454		C_13_H_12_O_8_	Caffeoylmalic acid	2.61 ± 0.01	8.09 ± 0.06	0.01 ± 0.01
6.21	193.0494		C_10_H_10_O_4_	Ferulic acid	0.05 ± 0.03	0.64 ± 0.03	0.02 ± 0.04
6.38	537.1972		C_26_H_34_O_12_	Tanegoside	0.48 ± 0.09	1.23 ± 0.08	ND
6.40	479.0825		C_21_H_20_O_13_	Caffeoylquinic acid hexoside	44.80 ± 0.08	24.47 ± 0.05	0.03 ± 0.03
				**Total identified**	**50.85** ± **0.12**	**45.05** ± **0.28**	**0.36** ± **0.06**

**Flavonoids**							
6.19	631.0938		C_28_H_24_O_17_	Myricetin 7-(6″-galloylglucoside)	3.96 ± 0.00	6.29 ± 0.01	ND
6.35	479.0826		C_21_H_20_O_13_	Myricetin glucoside	3.78 ± 0.01	4.57 ± 0.01	ND
6.40	433.0406	301.0347	C_19_H_14_O_12_	Quercetin-3-*O*-pentoside	1.93 ± 0.00	1.61 ± 0.00	0.04 ± 0.00
6.52	615.099		C_28_H_24_O_16_	Galloylquercetin	2.26 ± 0.01	2.99 ± 0.01	ND
6.60	449.0721		C_20_H_18_O_12_	Myricetin 3-arabinoside	2.56 ± 0.01	1.13 ± 0.01	ND
6.67	463.0876	301.0349	C_21_H_20_O_12_	Quercetin glucoside	18.16 ± 0.01	8.45 ± 0.01	1.21 ± 0.00
6.72	599.1036	285.0398	C_28_H_24_O_15_	Kaempferol galloyl hexoside	1.28 ± 0.01	1.16 ± 0.01	ND
6.83	477.1037		C_22_H_22_O_12_	Isorhamnetin 3-*O*-glucoside	0.65 ± 0.01	0.16 ± 0.00	ND
6.88	507.1139		C_23_H_24_O_13_	Syringetin-3-*O*-glucoside	0.41 ± 0.00	0.78 ± 0.01	ND
6.89	433.0768	301.0351	C_20_H_18_O_11_	Quercetin-3-*O*-pentoside	1.93 ± 0.00	1.61 ± 0.00	0.04 ± 0.00
6.99	447.0924	285.0403	C_21_H_20_O_11_	Kaempferol-3-*O*-hexoside	16.04 ± 0.01	9.34 ± 0.01	4.54 ± 0.00
7.00	301.0325		C_15_H_10_O_7_	Quercetin	7.31 ± 0.01	6.42 ± 0.01	0.28 ± 0.01
7.08	417.0814	285.0401	C_20_H_18_O_10_	Kaempferol 3-*O*-arabinoside	0.74 ± 0.01	0.47 ± 0.01	ND
7.20	625.1194	301.0352	C_30_H_26_O_15_	Quercetin-3-*O*-caffeoylhexoside	0.26 ± 0.01	0.50 ± 0.01	ND
7.29	431.0974	285.0401	C_21_H_20_O_10_	Kaempferol-7-*O*-rhamnoside	34.06 ± 0.01	12.63 ± 0.00	0.09 ± 0.00
7.45	609.1246		C_27_H_30_O_16_	Rutin	0.18 ± 0.01	0.28 ± 0.02	0.07 ± 0.00
7.80	301.0336		C_15_H_10_O_7_	Tricetin	8.26 ± 0.01	6.15 ± 0.09	0.30 ± 0.00
7.97	609.1248	301.0346	C_30_H_26_O_14_	Quercetin-*p*-coumaroylhexoside	0.07 ± 0.01	0.13 ± 0.01	0.05 ± 0.00
				**Total identified**	**103.85** ± **0.02**	**64.66** ± **0.11**	**6.65** ± **0.02**
1.05	173.0455	ND	C_7_H_10_O_5_	Shikimic acid	7.58 ± 0.15	2.27 ± 0.09	23.24 ± 0.05
4.94	295.1029	ND	C_11_H_20_O_9_	Rhamnosylarabinose	0.07 ± 0.05	0.02 ± 0.01	0.88 ± 0.03
				**Total identified**	**7.65** ± **0.17**	**2.29** ± **0.08**	**24.13** ± **0.05**
				**Total identified secondary metabolites**	**235.26** ± **0.39**	**227.92** ± **0.11**	**33.71** ± **0.09**

a ND: not detected.

Regarding the chemical classes of identified metabolites, the most representative in all the extracts were flavonoids (103 mg g^−1^, 65 mg g^−1^, and 7 mg g^−1^ in EA, MeOH, and water extracts, respectively). This predominance is in agreement with previous LC-MS-based metabolomic studies on *Epilobium angustifolium*, *E. parviflorum*, and *E. hirsutum*, where flavonol glycosides were consistently reported among the most abundant constituents of aerial parts.^9,56^ Within this chemical class, some metabolites already reported by other authors in *Epilobium* spp. were identified, such as several kaempferol and quercetin derivatives.^6,34,57^ These compounds are widely recognized as chemotaxonomic markers of the genus, and their occurrence has been consistently demonstrated across multiple species using LC-MS and NMR approaches.^58^ Several others are reported in this plant species for the first time. In particular, the detection of acylated flavonoids (*e.g.*, quercetin-caffeoylhexoside) and less common aglycones such as syringetin and tricetin derivatives suggests a broader flavonoid diversification compared to that previously described for *Epilobium* spp., where kaempferol, quercetin, and myricetin derivatives generally predominate. Kaempferol derivatives were the most representative (mainly kaempferol-3-*O*-hexoside, kaempferol 3-*O*-arabinoside, and kaempferol-7-*O*-rhamnoside), followed by quercetin glucoside. A similar dominance of kaempferol glycosides has been reported in *E. angustifolium* and *E. parviflorum*, although in those species quercetin derivatives are often found at comparable or higher levels, suggesting a possible species-specific flavonoid distribution in *E. dodonaei*.

Gallic acid derivatives were identified as the second most representative class of metabolites in *E. dodonaei* extracts. In this case, the highest extraction of these compounds was achieved using methanol instead of EA (116 mg g^−1^ and 73 mg g^−1^, respectively). Oenothein B, pedunculagin, galloyl glucose ellagic acid, and penta-*O*-galloyl-alpha-d-glucopyranose were the most representative constituents of this chemical class. Among these, oenothein B is considered the main ellagitannin of the genus *Epilobium*, often accounting for a large proportion of total polyphenols and widely used as a marker compound due to its abundance and biological relevance.^59^ The simultaneous detection of oenothein A, gemin D, sanguiin derivatives, and pedunculagin is in agreement with previous studies demonstrating that *Epilobium* species are characterized by complex oligomeric ellagitannins derived from hexahydroxydiphenoyl units.^60,61^ However, the relatively high amount of pedunculagin observed in the EtAc extract appears noteworthy, as in most reported *Epilobium* species this compound is present in lower concentrations compared to oenothein B, suggesting either a species-specific metabolic feature or a solvent-driven enrichment. The extraction yield of phenolic acids was comparable for EA (51 mg g^−1^) and MeOH (45 mg g^−1^). In this case, only 8 compounds were identified, with caffeoylquinic acid hexoside being the most representative. This finding is partially consistent with previous reports, in which chlorogenic acid and related caffeoylquinic derivatives are commonly detected in *Epilobium* spp., although typically at lower levels compared to ellagitannins.^62–65^ The relatively high abundance observed in the present study may therefore indicate a distinctive metabolic trait of *E. dodonaei*. This latter was also the most abundant compound identified in EtAc and MeOH extracts. Other phenolic acids, including caffeoylmalic acid, coumaric acid, and ferulic acid, were detected in agreement with previous phytochemical studies, where they are generally reported as minor constituents or intermediates in phenylpropanoid metabolism.

### 3.3 Antioxidant activity

Assessing the antioxidant properties of plant extracts could serve as a crucial guide to convert natural products in new potential drugs. In this sense, different extracts obtained from the aerial parts of *E*. *dodonaei* were investigated regarding their antioxidant properties, using different approaches, including the quenching of free radicals, reducing power, and the chelation of transition metals. The results are reported in Table 3. DPPH and ABTS radicals are commonly used to evaluate the radical-quenching abilities of plant extracts. In the tested extracts, the greatest radical scavenging ability was observed in methanol extract with 494.40 ± 1.22 mg TE g^−1^ for DPPH and 880.32 ± 7.83 mg TE g^−1^ for ABTS, followed by water extract with 490.86 ± 2.30 mg TE g^−1^ for DPPH and 845.06 ± 4.39 mg TE g^−1^ for ABTS. In both assays, ethyl acetate extracts showed the weakest scavenging ability on these radicals (47.08 ± 0.52 mg TE g^−1^ for DPPH and 77.04 ± 2.01 mg TE g^−1^ for ABTS). Reduction power assays assess the reduction of Cu^2+^ to Cu^+^ and Fe^3+^ to Fe^2+^ in the CUPRAC and FRAP assays, respectively. In the CUPRAC assay, the methanol extract showed the best ability with 881.03 ± 19.46 mg TE g^−1^, followed by the water extract with 832.36 ± 2.90 mg TE g^−1^. In the FRAP assay, the best reduction power was observed for the water extract with 521.71 ± 6.17 mg TE g^−1^ and for the methanol extract with 506.06 ± 14.39 mg TE g^−1^. Also, in these assays, the extract obtained in ethyl acetate showed the lowest reduction capacity (90.61 ± 0.96 mg TE g^−1^ for CUPRAC and 45.60 ± 0.61 mg TE g^−1^ for FRAP). Chelating transition metals is regarded as one of the important antioxidant mechanisms, as it can stop the generation of hydroxyl radicals during the Fenton reaction. In the present study, the best chelating ability was exhibited by the water extract with 25.96 ± 0.12 mg EDTA g^−1^, followed by the methanol extract with 25.52 ± 0.05 mg EDTA g^−1^, and but ethyl acetate was the weakest extract. The phosphomolybdenum reaction involves the reduction of Mo(vi) to Mo(v) by antioxidant compounds under acidic conditions. This assay has attracted interest in recent studies due to its simplicity and the lack of need for specific equipment. The methanol and ethyl acetate extracts demonstrated to be the best in this assay, with 3.48 ± 0.01 mmol TE g^−1^ and 3.47 ± 0.07 mmol TE g^−1^, respectively. The water extract was the weakest in this antioxidant assay (3.17 ± 0.05 mmol TE g^−1^). Combining all the antioxidant results, we can see that the methanolic and water extracts were the best, showing a correlation with the amount of phenols contained in the different extracts. In fact, these extracts were also the richest in terms of phenols. Previous studies^9,66^ on methanolic extracts of various *Epilobium* species, showed a strong presence of oenothein B, which is considered the primary compound responsible for the biological activities. Also, in our previous study on the relationship between the chemical components and biological properties of various extracts of aerial part of *E. hirsutum*, we found that oenothein B was the distinguished compound.^12^ Oenothein B is known to be efficacious antioxidants and the presence of this, especially in methanolic extracts, can explain the best antioxidant activity shown by these extracts, both in our previous study and in this one conducted on *E. dodonaei*.

**Table 3:** Antioxidant properties of the tested extracts^a^

Extracts	DPPH (mg TE g^−1^)	ABTS (mg TE g^−1^)	CUPRAC (mg TE g^−1^)	FRAP (mg TE g^−1^)	Chelating ability (mg EDTAE g^−1^)	Phosphomolybdenum (mmol TE g^−1^)
EA	47.08 ± 0.52^b^	77.04 ± 2.01^c^	90.61 ± 0.96^c^	45.60 ± 0.61^c^	9.07 ± 0.66^c^	3.47 ± 0.07^a^
MeOH	494.40 ± 1.22^a^	880.32 ± 7.83^a^	881.03 ± 19.46^a^	506.06 ± 14.39^b^	25.52 ± 0.05^b^	3.48 ± 0.01^a^
H_2_O	490.86 ± 2.30^a^	845.06 ± 4.39^b^	832.36 ± 2.90^b^	521.71 ± 6.17^a^	25.96 ± 0.12^a^	3.17 ± 0.05^b^

a Values expressed are means ± S.D. of three parallel measurements. EA: ethyl acetate extract, MeOH: methanol extract, H_2_O: water extract. TE: trolox equivalent; EDTAE: EDTA equivalent. na: not active. Different letters indicate significant differences in the tested extracts (*p* < 0.05).

### 3.4 Enzyme inhibitory properties

In recent times, there has been a steady rise in the prevalence of non-communicable global diseases, necessitating urgent measures to control their spread. In this context, researchers are actively exploring alternative and effective treatment strategies for managing these conditions. With the growing human population, synthetic compounds remain key components in these strategies.^67^ However, due to the toxic properties of most synthetic compounds, there is increasing interest in natural alternatives. Natural enzyme inhibitors, in particular, are becoming important in treating the aforementioned diseases. These natural compounds can inhibit the actions of enzymes linked to various chronic inflammatory and degenerative conditions.^68^ For this reason, natural compounds and extracts are usually tested against enzymes like α-amylase, α-glucosidase, tyrosinase, and cholinesterases, known also for their correlation with chronic diseases. In this study, different types of extracts derived from the aerial parts of *E. dodonaei* were tested for their inhibitory effects on cholinesterase, an enzyme deeply involved in Alzheimer's disease; tyrosinase, which is responsible for hyperpigmentation; and amylase and glucosidase, both of which are involved in type-2 diabetes. The results are reported in Table 4. Against AChE, the best inhibitory effect was observed for the methanol extract with a value of 2.18 ± 0.39 mg GALAE g^−1^, followed by the ethyl acetate extract, with a value of 1.55 ± 0.33 mg GALAE g^−1^, and water (0.80 ± 0.07 mg GALAE g^−1^). Also, for the tyrosinase inhibition, the order of ability was the same, with the best efficacy for the methanol extract with a value of 65.47 ± 1.65 mg KAE g^−1^, followed by ethyl acetate (43.89 ± 1.23 mg KAE g^−1^) and water (30.49 ± 0.93 mg KAE g^−1^). In the amylase inhibition assay, the best results were observed for ethyl acetate (0.39 ± 0.01 mmol ACAE g^−1^) and methanol (0.31 ± 0.01 mmol ACAE g^−1^). The water extract was the weakest with a value of 0.05 ± 0.01 mmol ACAE g^−1^. Regarding glucosidase inhibition, only the ethyl acetate extract was active with a value of 1.24 ± 0.06 mmol ACAE g^−1^. From these results, it's notable how water was the least active extract in every enzymatic inhibition assay. The total flavonoid content had a positive correlation with the tyrosinase inhibition; among flavonoids, kaempferol and quercetin were the most prominently found in the three extracts of the aerial part of the tested species. The tyrosinase inhibitory role of these two flavonoids has been reported several times in the literature.^69–71^

**Table 4:** Enzyme inhibitory properties of the tested extracts^a^

Extracts	AChE inhibition (mg GALAE g^−1^)	Tyrosinase inhibition (mg KAE g^−1^)	Amylase inhibition (mmol ACAE g^−1^)	Glucosidase inhibition (mmol ACAE g^−1^)
EA	1.55 ± 0.33^b^	43.89 ± 0.61^b^	0.39 ± 0.01^a^	1.24 ± 0.06
MeOH	2.18 ± 0.39^a^	65.47 ± 1.65^a^	0.31 ± 0.01^b^	na
H_2_O	0.80 ± 0.07^c^	30.49 ± 0.93^c^	0.05 ± 0.01^c^	na

a Values expressed are means ± S.D. of three parallel measurements. EA: ethyl acetate extract, MeOH: methanol extract, H_2_O: water extract. GALAE: galatamine equivalent; KAE: kojic acid equivalent; ACAE: acarbose equivalent. na: not active. Different letters indicate significant differences in the tested extracts (*p* < 0.05).

### 3.5 Skin-enzyme modulation by *Epilobium dodonaei*

**Fig. 2:**
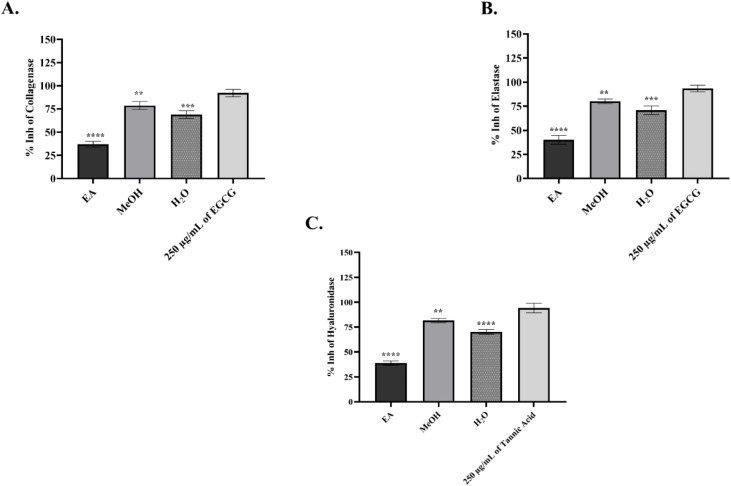
Inhibitory effects of *Epilobium dodonaei* extracts on skin-related enzymes. (A) % Inhibition of collagenase enzyme; (B) % inhibition of elastase enzyme; (C) % inhibition of hyaluronidase enzyme. EA: ethyl acetate extract, MeOH: methanol extract, H_2_O: water extract. 250 µg mL^−1^ EGCG was used as a positive control in panels A and B, and 250 µg mL^−1^ tannic acid was used in panel C. Data are presented as mean ± SD. Statistical significance was evaluated according to the respective control groups (**p < 0.01; ***p < 0.001; **p < 0.0001).

The inhibitory effects of *E. dodonaei* extracts on collagenase, elastase, and hyaluronidase are presented comparatively among the three extracts in Fig. 2. In the analyses performed, EGCG, a commercially available standard substance with known efficacy, was used for comparison in collagenase and elastase tests, while tannic acid was used for hyaluronidase (Fig. 2).^31^ In this context, the extracts with the highest inhibitory activity were ranked as follows: MeOH > H_2_O > EA. In collagenase inhibition (Fig. 2A), the MeOH extract showed the highest inhibition with 78.81%, followed by the H_2_O extract with 68.94%. The EA extract was the weakest extract with an inhibition rate of 37.09%. A similar pattern was observed in elastase inhibition; MeOH extract showed 80.16% inhibition, H_2_O extract 70.95%, and EA extract 40.14% (Fig. 2B). On the other hand, in the hyaluronidase test presented in Fig. 2C., it was determined that MeOH extract again exhibited the highest inhibition effect with 81.62%, while H_2_O extract provided the lowest inhibition with 70.27% and EA extract with 38.89%. This study demonstrates that *E*. *dodonaei* extracts possess a multifaceted regulatory potential on key biological processes associated with skin aging and inflammation. Chronic inflammation and oxidative stress form a common pathophysiological axis for many skin pathologies, including photoaging, epidermal barrier disruption, loss of dermal elasticity, and wound healing defects.^5,6^ Furthermore, it is reported in the literature that in these pathological processes, increased ROS and activation of transcription factors such as NF-κB/AP-1 accelerate ECM degradation, while excessive activity of enzymes such as collagenase, elastase, and hyaluronidase weakens dermal integrity and exacerbates signs of aging.^7,72^ Our results particularly highlight the MeOH extract's strong antioxidant capacity and significant enzyme inhibition. UPLC-QToF data in Table 1 and Fig. 1 confirm that the MeOH extract has a phenolic and ellagitannin-rich profile, including oenothein B, gallic acid derivatives, kaempferol, and quercetin glycosides. These compounds have previously been shown to be effective in dermal protective mechanisms such as ROS scavenging and enzyme inhibition.^12,73^ The potential of kaempferol and quercetin derivatives to regulate inflammatory signals and contribute to ECM stability is also supported by previous studies.^20,74,75^

This phytochemical diversity and information in the literature are consistent with the high inhibitory activity (78–82%) shown by the MeOH extract obtained in this study on collagenase, elastase, and hyaluronidase. The moderate inhibition of the water extract and the lower inhibition of the EA extract suggest that the extraction efficiency and polarity differences of the phenolic compounds determine the biological activity. Suppression of ECM degradation enzymes; it is known to have an effect that slows down aging associated with wrinkle formation, loss of elasticity and inflammation.^76^ When all findings were considered together, *E. dodonaei* emerges as a potent phytotherapeutic candidate capable of simultaneously targeting anti-aging, oxidative stress control, and ECM protective activities, particularly through polyphenolic compounds concentrated in the MeOH extract.^77^

### 3.6 Defining the non-cytotoxic concentration of *Epilobium dodonaei* extracts by WST-1

**Fig. 3:**
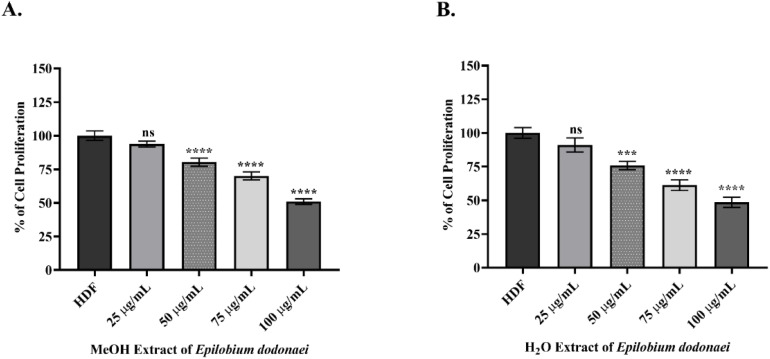
Effects of *Epilobium dodonaei* extracts on human dermal fibroblast (HDF) cell proliferation by WST-1 analysis. (A) Effect of MeOH extract at concentrations of 25–100 µg mL^−1^ on HDF cell viability. (B) Proliferation profile of H_2_O extract in the same concentration range. The control group (HDF) was considered to have 100% viability. Data were presented as mean ± SD. Statistical significance was evaluated using GraphPad Prism software compared to the control group (ns: *p* > 0.05; ***p < 0.001; ****p < 0.0001).

In Fig. 3, the effect of MeOH and H_2_O extracts on human dermal fibroblast viability was evaluated at 48 hours using the WST-1 assay. The control group (untreated HDF) was accepted as the 100% viability reference, and calculations were made based on untreated HDF.^31^

In this context, while the concentration of MeOH extract at 25 µg mL^−1^ did not cause a significant decrease in cell viability (93.78%; ns), viability gradually decreased at increasing concentrations and was measured as 80.31% at 50 µg mL^−1^ (*P* ≤ 0.0001 for ****), 70.04% at 75 µg mL^−1^ (*P* ≤ 0.0001 for ****), and 50.95% at 100 µg mL^−1^ (*P* ≤ 0.0001 for ****) (in Fig. 3A). A similar cellular response was observed in the H_2_O extract. A concentration of 25 µg mL^−1^ did not significantly alter viability (91.06%; ns). At higher concentrations, viability decreased to 75.77% at 50 µg mL^−1^ (*P* ≤ 0.001 for ***), 61.22% at 75 µg mL^−1^ (*P* ≤ 0.0001 for ****), and 48.51% at 100 µg mL^−1^ (*P* ≤ 0.0001 for ****) (in Fig. 3B).

The results presented in Fig. 3 demonstrate that *E. dodonaei* MeOH and H_2_O extracts are well tolerated in terms of fibroblast viability at 48-hours exposure, which is thought to be due to the high total phenolic content (MeOH: 145 mg GAE g^−1^; H_2_O: 141 mg GAE g^−1^) reported in Table 2 and the specific compounds identified by UPLC-QToF. The ellagitannins (especially oenothein B and pedunculagin), gallic acid derivatives, kaempferol, and quercetin glycosides contained in the MeOH extract have been reported in the literature to exhibit both low cytotoxicity and inflammation-suppressing properties in fibroblasts.^20,73,75^ Oenothein B, in particular, plays a leading role in the biological activity of *Epilobium* species due to its inhibitory properties on 5-α-reductase and aromatase, its ability to suppress COX-mediated inflammation, and its strong antioxidant capacity.^22,61^ Furthermore, kaempferol and quercetin derivatives have been shown to reduce oxidative stress-induced inflammatory responses in fibroblasts and maintain cell viability.^78^ The high presence of these compounds in MeOH and H_2_O extracts enhances the antioxidant/anti-inflammatory effect in both extracts and supports the non-toxic concentration range, consistent with our WST-1 results. In contrast, although the EA extract had the highest total metabolite content in the UPLC-QToF data (235 mg g^−1^), its lower total phenolic value (35 mg GAE g^−1^) in Table 2 indicated weaker antioxidant capacity and consequently lower efficacy in skin enzyme inhibition. It has also been reported in the literature that lipophilic *Epilobium* fractions can exhibit a more pronounced antiproliferative effect in fibroblasts when their phenolic density is low.^79^ Therefore, the low performance of the EA extract in enzyme results and its weaker activity in the inflammation/anti-aging axis make its non-use in future *in vitro* models scientifically rational. In conclusion, *E. dodonaei* MeOH and H_2_O extracts can be safely used at a working concentration of 75 µg mL^−1^ due to their high phenolic content, strong ellagitannin-flavonoid profile, and low cytotoxicity, and represent the most suitable phytochemical fractions for experiments targeting mechanisms associated with dermal inflammation and skin aging.

### 3.7 Alterations in the LPS-mediated inflammatory protein pathway following *Epilobium dodonaei* administration

**Fig. 4:**
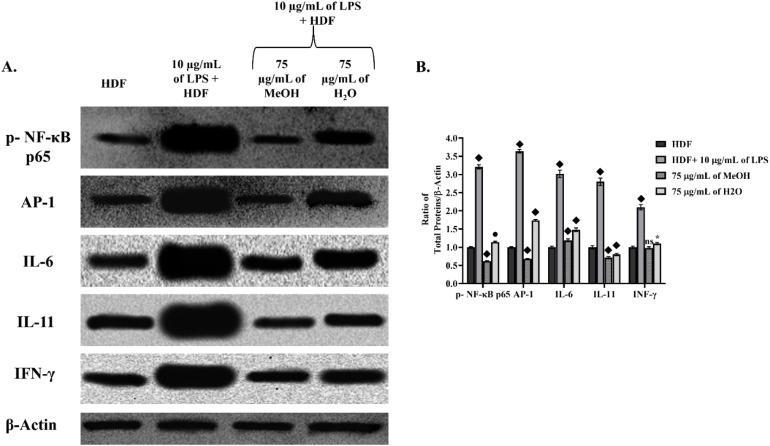
Western blot analysis of the effects of *Epilobium dodonaei* extracts on LPS-induced inflammatory response. (A) Band density images of membranes visualized with ECL obtained using the ChemiDoc system. Expression levels of p-NF-κB p65, AP-1, IL-6, IL-11, and IFN-γ proteins were evaluated; β-Actin was used as a loading control. (B) Statistical evaluation results obtained by quantitatively analyzing the relevant bands normalized to β-Actin. It was shown that inflammation markers, which were significantly increased with LPS application, were significantly decreased with both MeOH and H_2_O extract application (* indicated the *p* < 0.05; ● * indicated the *p* < 0.01; ◆ * indicated the *p* < 0.0001; ns: insignificant).

Skin inflammation is a biological process that begins with the activation of transcription factors, particularly NF-κB and AP-1, followed by the overproduction of pro-inflammatory cytokines such as IL-6, IL-11, and IFN-γ. Since these proteins are key determinants of dermal inflammation, this inflammatory pathway was targeted in LPS-induced HDF cells in this study; subsequently, the regulatory effects of *E. dodonaei* MeOH and H_2_O extracts at a study concentration of 75 µg mL^−1^ on these proteins were evaluated (in Fig. 4). All values were normalized to β-actin, and the untreated HDF group was used as the 1-fold reference (in Fig. 4B). NF-κB (p-NF-κB p65) levels increased 3.21-fold (*P* ≤ 0.0001 for ♦) with LPS application, indicating the onset of an inflammatory response. In contrast, p-NF-κB levels decreased by 0.62-fold in LPS-induced HDF cells treated with 75 µg mL^−1^ MeOH extract (*P* ≤ 0.0001 for ♦). The effect of H_2_O extract similarly reduced p-NF-κB levels to 1.14-fold (*P* ≤ 0.01 for ●). AP-1 protein levels increased to 3.64-fold (*P* ≤ 0.0001 for ♦) in the LPS group. MeOH extract significantly reduced AP-1 levels (0.68-fold; *P* ≤ 0.0001 for ♦); H_2_O extract resulted in a decrease of up to 1.74-fold (*P* ≤ 0.0001 for ♦). Similarly, IL-6 protein levels were found to be significantly increased (3.02-fold; *P* ≤ 0.0001 for ♦) after LPS application to HDF cells. While MeOH extract reduced IL-6 levels to 1.19-fold (*P* ≤ 0.0001 for ♦), H_2_O extract was observed to reduce IL-6 levels to 1.48-fold (*P* ≤ 0.0001 for ♦) after application.

The inflammatory response induced by LPS resulted in a significant increase not only in early transcription factors but also in the levels of later response components, IL-11 and IFN-γ proteins. Specifically, IL-11 protein levels increased to 2.80-fold *P* ≤ 0.0001 for ♦) in the LPS group; MeOH extract significantly suppressed this increase, reducing it to 0.71-fold (*P* ≤ 0.0001 for ♦). H_2_O extract showed a similar trend, reducing IL-11 to 0.80-fold (*P* ≤ 0.0001 for ♦) and decreasing the inflammatory load. IFN-γ protein levels, representing later stages of inflammation, also increased significantly with LPS (2.10-fold; *P* ≤ 0.0001 for ♦). MeOH extract provided the strongest inhibition of IFN-γ, reducing its level by up to 0.98-fold (ns), thereby eliminating the inflammation parameter caused by LPS by reducing it to untreated HDF levels and thus restoring a healthy HDF profile. It was also observed that H_2_O extract reduced IFN-γ protein levels by 1.14-fold following application (*).

LPS triggers inflammatory signaling by interacting with Toll-like receptor 4 (TLR4) on the cell surface, initiating a chain that activates key transcription factors such as NF-κB (nuclear factor kappa B) and AP-1. This stimulus leads to the release of inactive NF-κB in the cytoplasm through phosphorylation of p65/p50 heterodimers bound to the IκB (inhibitory kappa B) protein and ubiquitination-dependent degradation of IκB. The released NF-κB complexes translocate to the nucleus, increasing the gene expression of pro-inflammatory cytokines such as IL-6, IL-11, and IFN-γ.^80,81^ This mechanism, which has been clarified in the literature, supports the increase in NF-κB and AP-1 protein levels observed in LPS-stimulated HDF cells in our study. Normally, NF-κB, which is inactive and bound to IκB in the cytoplasm, is released and passes into the nucleus when the IKK complex phosphorylates and breaks down IκB.^82^ AP-1 activation is also triggered by stimuli such as TNF-α and LPS *via* MAPK signaling pathways, supporting the production of inflammatory cytokines.^82,83^ The phytochemical analysis results obtained within the scope of our study, presented in Table 1, revealed that the MeOH and H_2_O extracts are rich in polycyclic phenolics (especially oenothein B and gallic acid derivatives) and flavonoids (kaempferol and quercetin glycosides). It has been previously shown that these compounds can reduce the production of cytokines.^20,24^ On the other hand, it has been found that LPS not only leads to an increase in inflammatory proteins such as NF-κB and AP-1, but also disrupts the oxidative stress balance by triggering the overproduction of intracellular ROS.^84,85^ This situation has been described in the literature as LPS causing an inflammation-oxidative stress cycle that begins with TLR4 activation, increases mitochondrial ROS, accelerates the phosphorylation of NF-κB, and creates positive feedback in pro-inflammatory cytokines.^78,86,87^ Considering this mechanism, the suppression of NF-κB/AP-1 axis upregulation by MeOH and H_2_O extracts in our study can be explained not only by directly reducing the inflammatory response but also by the high total phenolic content presented in Table 2 and the strong total antioxidant capacities reported in Table 4. Phenolic compounds, particularly ellagitannins (oenothein B), gallic acid derivatives, kaempferol, and quercetin glycosides, are primary phytochemicals contributing to both ROS scavenging capacity and suppression of ROS-mediated NF-κB activation.^24,88^ The higher total phenolic content and antioxidant capacity observed in the MeOH extract provide the biochemical basis for the decrease in NF-κB and AP-1 protein levels. This is because the reduction in ROS levels suppresses the overphosphorylation of IκB and thus the translocation of NF-κB to the nucleus; thereby reducing the production of downstream cytokines such as IL-6, IL-11, and IFN-γ.^80^ The H_2_O extract exhibiting a more moderate but significant anti-inflammatory effect is consistent with the phenolic component and antioxidant capacity values in Tables 2 and 4. Neutralization of ROS by phenolic compounds is one of the key mechanisms contributing to the suppression of inflammatory pathways. This study demonstrates for the first time that MeOH and H_2_O extracts of *E*. *dodonaei* can simultaneously target LPS-induced ROS imbalance and increased inflammatory proteins. In this respect, the therapeutic potential of the plant in dermal inflammation and ROS-induced cellular aging models is scientifically supported.

### 3.8 Effects of *E. dodonaei* extracts on MMP-2 and MMP-9 enzyme activity and gene levels

**Fig. 5:**
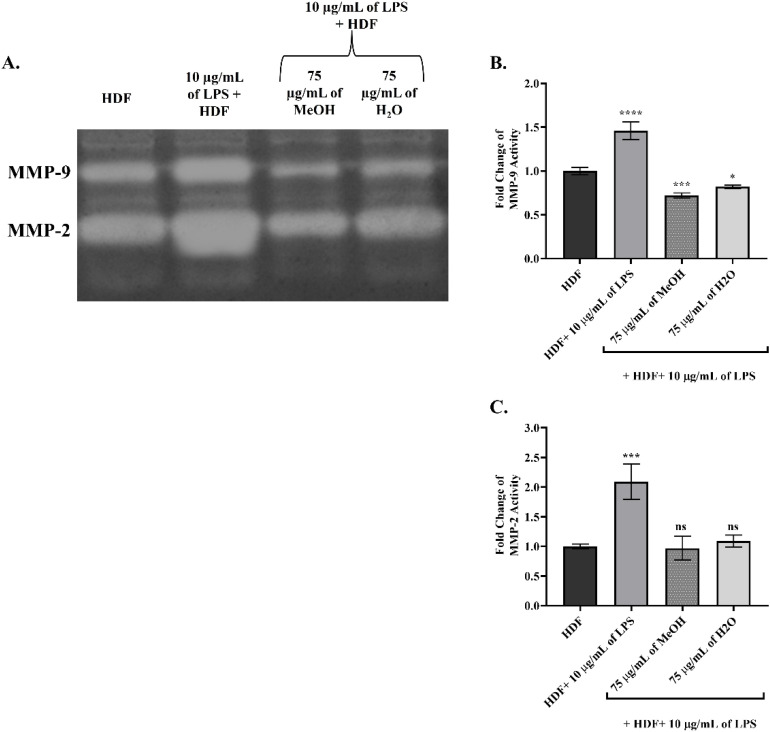
Evaluation of the effects of *Epilobium dodonaei* extracts on MMP-2 and MMP-9 enzymatic activity in LPS-induced HDF cells using gelatin zymography. (A) Lytic white images of MMP-2 and MMP-9 bands obtained from zymography performed on polyacrylamide gel containing gelatin. (B) Quantitative analysis of MMP-9 band intensities and fold-change representation compared to the control group. (C) Quantitative analysis of MMP-2 band intensities and fold-change representation compared to the control group. Data were presented as mean ± SD. Statistical significance was evaluated according to the LPS group (*p < 0.05; ***p < 0.001; ****p < 0.0001; ns: insignificant).

**Fig. 6:**
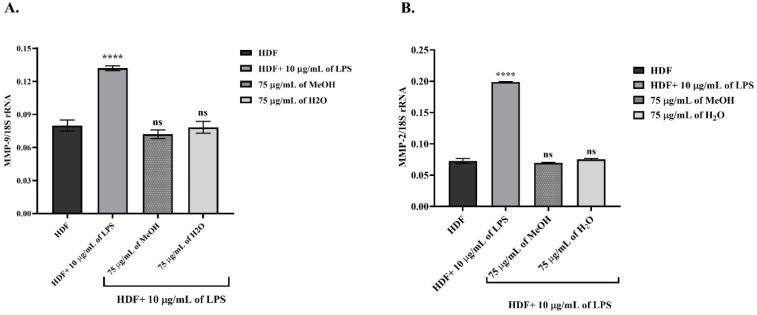
RT-PCR analysis of the effects of *Epilobium dodonaei* extracts on MMP expression in LPS-induced HDF cells. (A) Normalized levels of MMP-9 gene expression to 18S rRNA. (B) Normalized levels of MMP-2 gene expression to 18S rRNA. Data were presented as mean ± SD. Statistical significance was evaluated according to the LPS group (****p < 0.0001; ns: insignificant).

The main determinants of ECM integrity, MMP-2 and MMP-9, were evaluated in LPS-induced HDF cells in terms of both zymography band intensity (in Fig. 5) and RT-PCR gene level (in Fig. 6). Increased MMP enzyme activity was associated with the degradation of the gelatin substrate, and the gel image showing increased band intensity indicating higher MMP activity is presented in Fig. 5A. Comparative statistical data, using a 1-fold increase in enzyme activity in untreated HDF, was presented in Fig. 5B. Furthermore, in Fig. 6, 18S rRNA was used as the housekeeping gene in RT-PCR analysis to detect changes in gene expression, and all MMP gene levels were normalized to this reference.

In LPS-induced HDF cells, a significant increase in MMP-9 enzyme activity was observed. MMP-9 activity, which was 1.00-fold in the untreated HDF group, increased to 1.46-fold (*P* ≤ 0.0001 for ****) after LPS application, indicating increased ECM degradation. On the other hand, MeOH application reduced enzyme activity by 0.72-fold (*P* ≤ 0.001 for ***), and similarly, H_2_O extract application resulted in a decrease of up to 0.82-fold activity, as shown in Fig. 5A. Similar findings were observed in MMP-2 activity; enzyme activity increased 2.09-fold (*P* ≤ 0.001 for ***) in LPS-induced HDF cells, while these levels decreased to 0.97- (ns) and 1.09-fold (ns), respectively, following the application of MeOH and H2O in Fig. 5B.

Similarly, in Fig. 6, which includes gene expression results, the MMP-9 mRNA level was 0.079 in untreated HDF cells, while LPS application increased this value to 0.132 (*P* ≤ 0.001 for ****). This indicates that LPS strongly induces MMP-9 transcription. MeOH extract application reduced the MMP-9 mRNA level to 0.072 (ns), bringing expression closer to basal levels. H_2_O extract provided similar suppression with a value of 0.077 (ns). These findings demonstrate that both extracts completely suppressed the LPS-induced MMP-9 gene increase and exerted an ECM-protective effect at the transcriptional level. In untreated HDF cells, MMP-2 mRNA levels, which were 0.072, increased to 0.198 (*P* ≤ 0.0001 for ****) with LPS, revealing that the MMP-2 gene is significantly induced under inflammation. MeOH extract significantly reduced MMP-2 expression to 0.065 (ns). H_2_O extract showed a similar effect, suppressing it to 0.075 (ns). RT-PCR results paralleled zymographic analysis, showing that both extracts reversed the MMP-2 transcription, which was increased by LPS, back to basal levels, thus restoring ECM stability at the molecular level.

The results obtained in this study show a significant increase in MMP-2 and MMP-9 enzyme activities (zymography) and mRNA levels (RT-PCR, normalized to 18S rRNA) in LPS-stimulated HDF cells, indicating disruption of ECM integrity and that gelatinases are important targets in the inflammatory response. The literature states that LPS increases mitochondrial ROS production *via* TLR4, and that increased ROS activates the p38/MAPK-NF-κB/AP-1 pathways, thereby increasing MMP-9 and MMP-2 expression and accelerating ECM degradation.^89^ In line with this mechanism, our study showed that LPS increased MMP-9 activity by approximately 1.5-fold and MMP-2 activity by more than 2-fold; RT-PCR data also revealed significantly elevated transcript levels of both genes. This indicates that increased proteolytic activity disrupts ECM structure and dermal hemostasis.^90^ The fact that MMP-9 and MMP-2 activities decreased significantly below the LPS group in zymography after application of MeOH and H_2_O extracts, and that mRNA levels returned to almost basal HDF levels in RT-PCR data, indicates that *E. dodonaei* extracts can restore ECM homeostasis by inhibiting ECM degradation at the molecular level. The MeOH extract reduced MMP-9 activity by more than half, while bringing MMP-2 to control levels; the H_2_O extract provided a more moderate but similar improvement. This bidirectional enzyme and gene suppression suggests that not only is LPS-driven overexpression of MMPs inhibited, but also upstream signaling pathways are modulated.^91^ The phenolic profile presented in Table 1 supports these results from a biochemical perspective. Ellagitannins (especially oenothein derivatives), ellagic acid/urolithin metabolites, kaempferol, and quercetin glycosides, which are abundant in the MeOH extract, have been identified as compounds with both ROS scavenging and MMP regulatory effects *via* NF-κB/AP-1. Various models have shown that ellagitannins and urolithins reduce MMP-9 and pro-inflammatory cytokines by suppressing NF-κB and ERK1/2 signaling; while kaempferol and quercetin reduce MMP-2/-9 expression and activity by decreasing AP-1 and NF-κB binding activity.^92–96^ In conclusion, these data demonstrate that E. dodonaei extracts can suppress the increased MMP-2/MMP-9 enzyme and gene levels resulting from LPS-induced ROS imbalance and inflammatory signaling at both functional (zymography) and transcriptional (RT-PCR) levels, thus restoring the balance of disrupted ECM structure and homeostasis; strongly supporting the plant's potential as a phytotherapeutic candidate in pathologies associated with dermal inflammation and ECM degradation.

### 3.9 Scavenging of LPS-induced cellular oxidation by application of *E. dodonaei* extracts

**Fig. 7:**
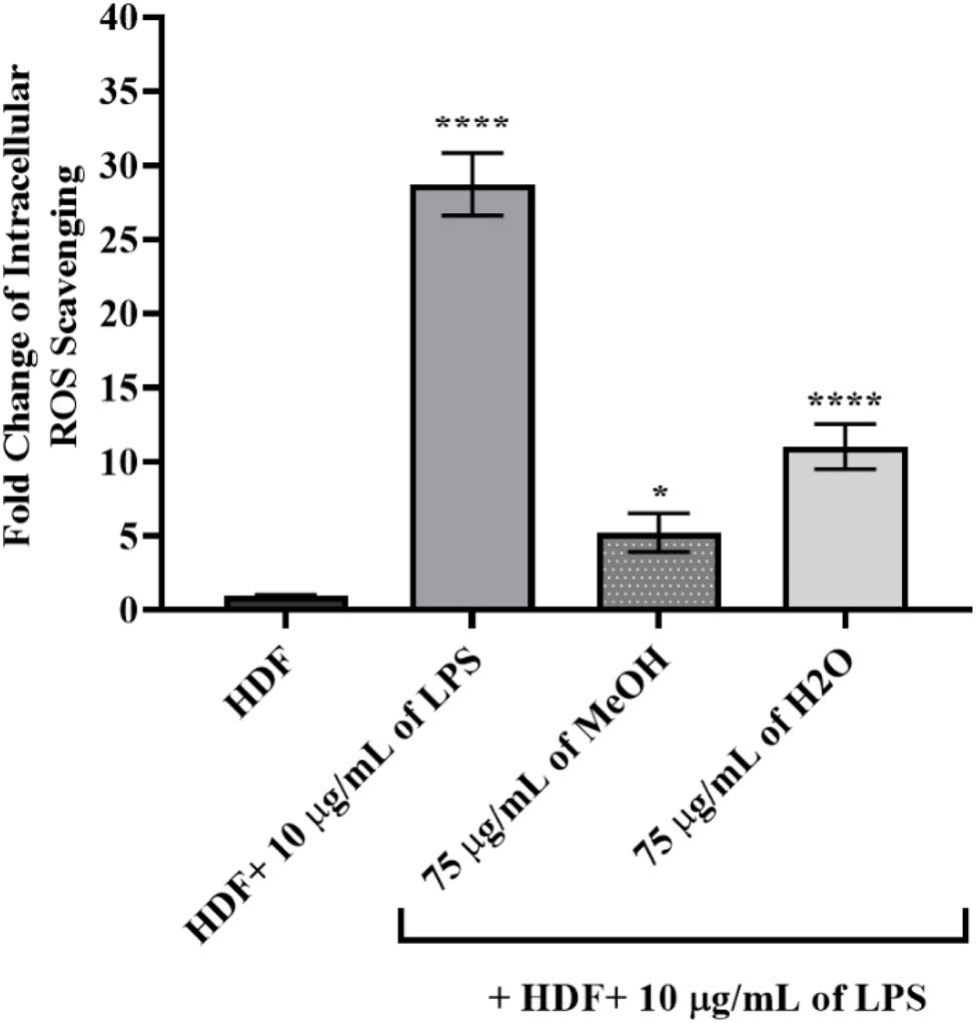
Evaluation of the effects of *Epilobium dodonaei* extracts on intracellular ROS levels in LPS-induced HDF cells using DCFDA analysis. Data were presented as mean ± SD. Statistical significance was evaluated according to the LPS group (*p < 0.05; ****p < 0.0001).

In Fig. 7, intracellular ROS levels in LPS-stimulated HDF cells were significantly increased, reaching 28.74-fold (*P* ≤ 0.0001 for ****) compared to the untreated HDF group. A concentration of 75 µg mL^−1^ of the MeOH extract significantly reduced ROS levels by 5.22-fold (*P* ≤ 0.05 for *), while the H_2_O extract provided an even stronger reduction, reaching 11.05-fold (*P* ≤ 0.0001 for ****). These findings demonstrate that both extracts reduce LPS-induced oxidative stress, with the MeOH extract exhibiting a stronger ROS scavenging capacity due to its higher phenolic content. The elevated ROS levels observed in LPS-induced HDF cells are consistent with the mechanism described in the literature, where TLR4-mediated mitochondrial ROS production increases oxidative load by activating inflammatory signaling pathways (NF-κB, AP-1, MAPK). Numerous studies have shown that LPS increases NADPH oxidase activation as an early phase reaction, that increased ROS enhances NF-κB phosphorylation, and thus creates a positive feedback loop in inflammatory cytokine and MMP expression.^97–99^ This study demonstrates for the first time that MeOH and H_2_O extracts of *E. dodonaei* significantly reduce excessive ROS accumulation caused by LPS. The reduction of ROS levels by approximately 5.22-fold for the MeOH extract and 11.05-fold for the H_2_O extract is directly related to the plant's phytochemical profile. Tables 1 and 2 show that the total phenolic content and flavonoid content of the MeOH extract are significantly higher than those of the H_2_O extract. Phenolic compounds—particularly ellagitannins (oenothein derivatives), gallic acid, ellagic acid, kaempferol, and quercetin glycosides—possess strong antioxidant mechanisms such as direct neutralization of reactive oxygen species, metal chelation, and suppression of NADPH oxidase activity.^74,100–102^ The high DPPH, ABTS, and FRAP values shown in Table 3 support the idea that the MeOH extract, in particular, possesses a strong electron-donor capacity and that its phenolic density is reflected in its biological ROS scavenging activity. Ellagitannins have been shown in various experimental models to disrupt the ROS-inflammation cycle by inhibiting NF-κB and AP-1 activation, thanks to their antioxidant and anti-inflammatory properties.^103,104^ In parallel with this mechanism, the decrease in ROS levels in our study, consistent with our previous results, was observed along with a decrease in NF-κB, AP-1, IL-6, IL-11, and IFN-γ protein levels, as well as a decrease in MMP-2/9 activity and gene levels, confirming that ROS is the main trigger of the inflammatory and matrix-destructive response. Reducing the ROS load with extract applications suppressed upstream signaling pathways, thus significantly improving both inflammation and ECM degradation.

In conclusion, it has been scientifically demonstrated that phenolic-rich extracts of *E. dodonaei* suppress LPS-induced oxidative stress, reducing both inflammatory protein levels and MMP activity; this is related to the plant's antioxidant capacity and polyphenolic components. This study demonstrates that *E. dodonaei* is a strong phytotherapeutic candidate that can be evaluated in pathologies accompanied by dermal inflammation, oxidative damage, and ECM disruption.

### 3.10 Molecular modeling results

#### 3.10.1 Binding energy

**Fig. 8:**
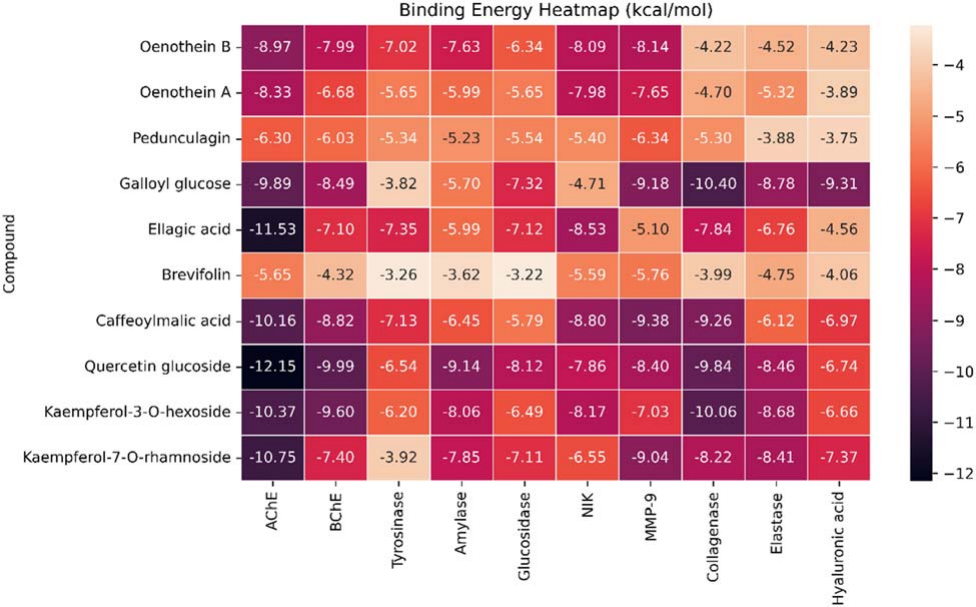
Heatmap of binding energies (kcal mol^−1^) for selected polyphenolic compounds across multiple enzyme targets. More negative values indicate stronger binding affinity, with −7 kcal mol^−1^ as a reference threshold for favorable interactions. Docking was performed for acetylcholinesterase (AChE), butyrylcholinesterase (BChE), tyrosinase, α-amylase, glucosidase, NF-κB inducing kinase (NIK), matrix metalloproteinase-9 (MMP-9), collagenase, elastase, and hyaluronidase. Oenothein B, oenothein A, and Pedunculagin were docked into allosteric pockets of the enzymes using SwissDock with attracting cavities, while the remaining compounds were docked into active sites using AutoDock 4.2.6.

The molecular docking analysis revealed distinct binding behaviors between compounds docked into the allosteric site (oenothein B, oenothein A, and pedunculagin *via* SwissDock with attracting cavities^47^)—due to their bulkiness that made them unfit for the enzyme's active site—and those docked into the active site of the respective enzymes (Fig. 8). Among the allosteric binders, oenothein B exhibited the strongest affinity toward AChE (−8.97 kcal mol^−1^), NIK (−8.09 kcal mol^−1^), and MMP-9 (−8.14 kcal mol^−1^). Oenothein A displayed slightly weaker binding across targets, while pedunculagin demonstrated generally lower affinities, suggesting reduced efficacy in the allosteric pocket. Allosteric binding, which can modulate enzymatic activity without directly competing with substrates, may confer advantages in selectivity and reduced off-target effects.^105^ In contrast, active site docking produced overall stronger interactions, with quercetin glucoside showing the highest affinity for AChE (−12.15 kcal mol^−1^) and strong multi-target activity against BChE (−9.99 kcal mol^−1^) and glucosidase (−8.12 kcal mol^−1^). Ellagic acid (−11.53 kcal mol^−1^ for AChE) and kaempferol glycosides (−10.37 to −10.75 kcal mol^−1^ for AChE) also ranked among the most potent inhibitors. Polyphenolic compounds such as these possess abundant hydroxyl groups capable of hydrogen bonding and π–π stacking, which likely contribute to their high docking scores.^106^ Notably, caffeoylmalic acid demonstrated strong inhibition potential for both AChE (−10.16 kcal mol^−1^) and MMP-9 (−9.38 kcal mol^−1^), while galloyl glucose combined good AChE affinity (−9.89 kcal mol^−1^) with potent MMP-9 binding (−9.18 kcal mol^−1^). These findings indicate that active site ligands generally displayed stronger predicted affinities than allosteric binders, consistent with the competitive inhibition mechanism associated with catalytic pocket targeting.^107^

Galloyl glucose exhibits consistently strong binding toward all three extracellular matrix-degrading enzymes, with a clear preference for hyaluronidase. Galloyl glucose binds collagenase with a markedly favorable binding energy (−10.40 kcal mol^−1^), indicating high affinity likely driven by extensive hydrogen bonding and aromatic interactions within the active-site cleft. Its interaction with elastase is moderately strong (−8.78 kcal mol^−1^), reflecting a more compact binding mode compatible with the enzyme's shallower pocket. Notably, galloyl glucose shows its most favorable binding toward hyaluronidase (−9.31 kcal mol^−1^), comparable to or stronger than many flavonoid glycosides, consistent with substrate-mimetic recognition of its glucose moiety and additional stabilization from the galloyl group. Overall, the broad-spectrum activity observed for certain flavonoids and tannins suggests potential for multitarget therapeutic strategies in complex diseases involving cholinesterase, glycosidase, and kinase pathways.^108^

### 3.11 Protein–ligand interaction

**Fig. 9:**
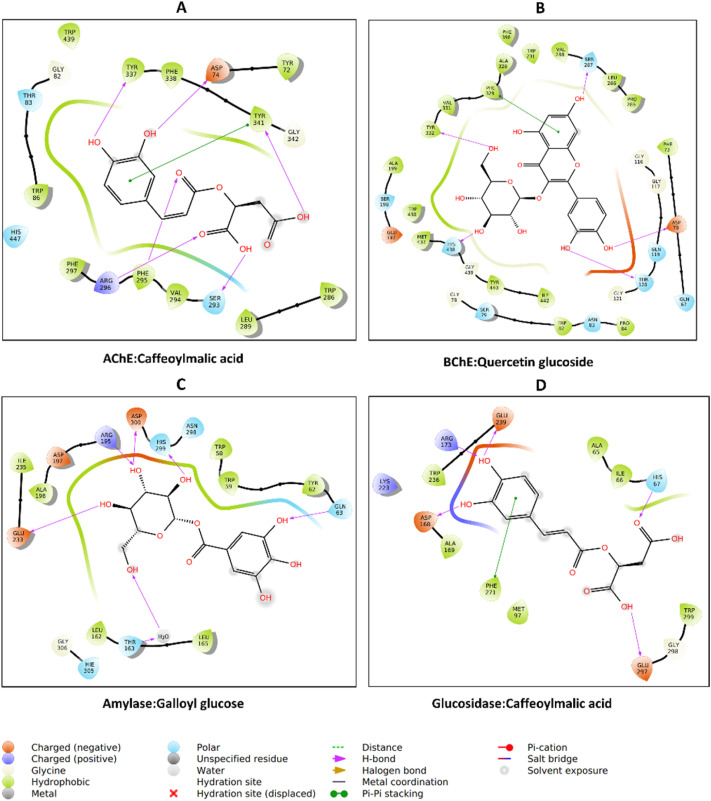
Protein–ligand interaction: (A) AChE_caffeoylmalic acid, (B) BChE_quercetin glucoside, (C) amylase_galloyl glucose, and (D) glucosidase_caffeoylmalic acid.

Caffeoylmalic acid is stabilized in the AChE active site through multiple hydrogen bonds with residues such as Asp74, Ser293, Phe295, Arg296, and Tyr337, providing strong directional interactions that anchor the molecule in place. Hydrophobic contacts are observed with residues like Trp286, Leu289, Val294, and Phe338, which help position the ligand in a nonpolar environment and reduce solvent exposure. Additionally, π–π stacking (pink lines) between the ligand's aromatic ring and residues such as Tyr341 enhances binding specificity (Fig. 9A). The observed H-bond with key residue Phe295 and π-stacking hydrophobic contact with Trp286 may play crucial roles in AChE inhibition.^109^ This combination of polar and nonpolar interactions suggests a well-optimized ligand fit, which is crucial for both stability and affinity within the active site.

Quercetin glucoside is primarily surrounded by hydrophobic residues, including Ala199, Trp321, Ala328, Phe330, and Val331. Several hydrogen bonds are formed with Asp70, Thr120, Ser287, Tyr332, and His438, anchoring the ligand's hydroxyl groups to the protein's polar residues. Charged residues, such as Ser198, Gln67, Asn83, and Gln119 around the periphery engage in van der Waals interaction (Fig. 9B). His438 is a member of the catalytic triad of BchE; hence, its blockade may contribute to the enzyme's inhibition.^110^ The aromatic core of the ligand fits into a largely hydrophobic cavity, suggesting that shape complementarity and van der Waals contacts play a major role in stabilizing this complex, alongside hydrogen bonds.

Caffeoylmalic acid interacts extensively through hydrogen bonds with residues such as Gln63 and Thr163 near the entrance to the amylase catalytic channel, and Arg195, Glu233, His299, and Asp300 deep inside the tunnel *via* its multiple hydroxyl groups and polar functional moieties. Hydrophobic contacts with residues that include Trp58, Trp59, Leu165, and Ala198 help to secure the ligand within a nonpolar sub-pocket, contributing to the entropic stability of the complex (Fig. 9C). Trp59 plays a key role in the stabilization of glucose moieties in the catalytic channel of amylase.^111^

Similarly, caffeoylmalic acid forms interactions with residues such as His67 and Glu297 near the gate of the glucosidase active site channel, and Asp168, Arg173, and Glu239 deep inside, which align polar groups for optimal binding geometry. A π-cation interaction is observed between the ligand's aromatic ring and Phe271, which adds a strong electrostatic stabilization. Hydrophobic residues, including Ala65, Ala166, Met97, and Trp236, create a nonpolar environment around the ligand's aromatic (Fig. 9D). Thus, the presence of both hydrophobic packing and π–π stacking interactions, combined with targeted hydrogen bonds, suggests that this binding mode is both geometrically tight and energetically favorable.

**Fig. 10:**
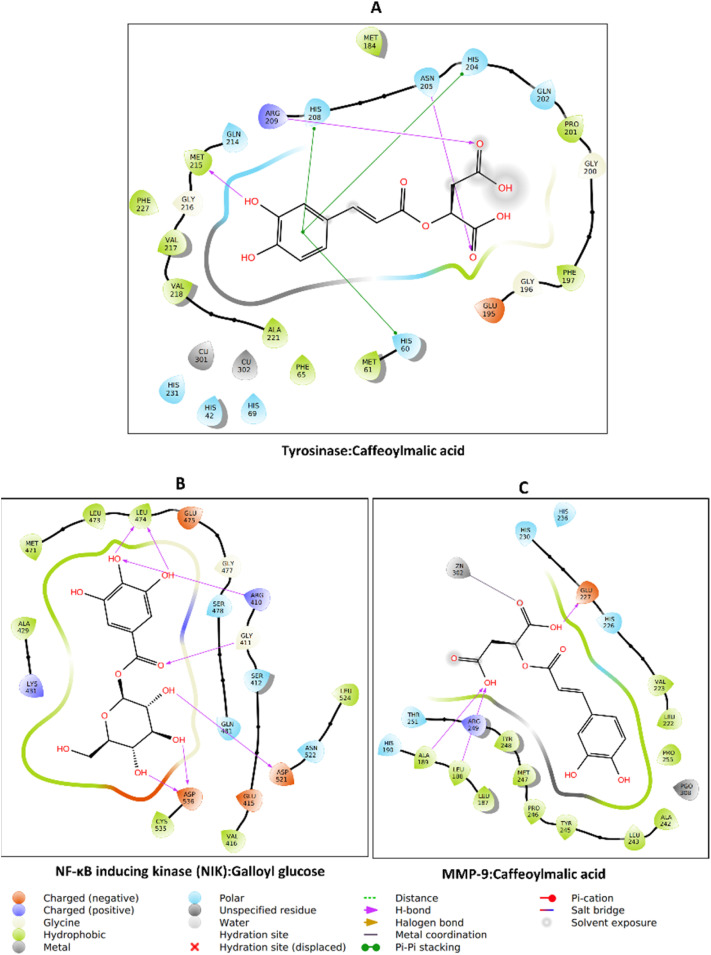
Protein–ligand interaction: (A) tyrosinase_caffeoylmalic acid, (B) NF-κB inducing kinase (NIK)_galloyl glucose, and (C) MMP-9_caffeoylmalic acid.

Caffeoylmalic acid forms multiple stabilizing interactions within the tyrosinase active site (Fig. 10A). Key hydrogen bonds are established between hydroxyl groups of the ligand and Asn205 and Arg209, while additional contacts are formed with Met271. Hydrophobic interactions are observed with residues such as Phe197, Val217, Val218, and Ala221, helping to anchor the ligand. In addition, π–π stacking interactions are formed between the aromatic head of the ligand and His60, His204, and His208. His60 and His208 are critical for the enzyme activity.^112^ Notably, metal coordination with the active site copper ions (Cu301, Cu302) suggests direct involvement in inhibiting the catalytic center, consistent with known tyrosinase inhibition mechanisms.^113^

Galloyl glucose engages in extensive hydrogen bonding with residues Arg410, Gly411, Asp521, Asp536, and the backbone of Leu4744, supporting a strong polar interaction network within the kinase pocket. Hydrophobic contacts with residues like Val416, Ala429, and Leu473 contribute to ligand orientation. The multiple van der Waals interactions formed serve to enhance binding (Fig. 10B). The binding mode indicates that galloyl glucose may block ATP-binding or substrate-recognition regions, potentially modulating NF-κB activation pathways.^114^ Caffeoylmalic acid is stabilized in the MMP-9 active site through conventional hydrogen bonding with Glu227, and with Leu188 and Ala189 *via* the backbone. Hydrophobic residues such as Val223, Ala242, Leu243, and Pro255 interact with the aromatic backbone of the ligand, while zinc ion coordination (Zn302) is observed (Fig. 10C), implicating direct interference with the catalytic metalloproteinase core. Such metal chelation is a known mechanism for MMP inhibition.^115^

**Fig. 11:**
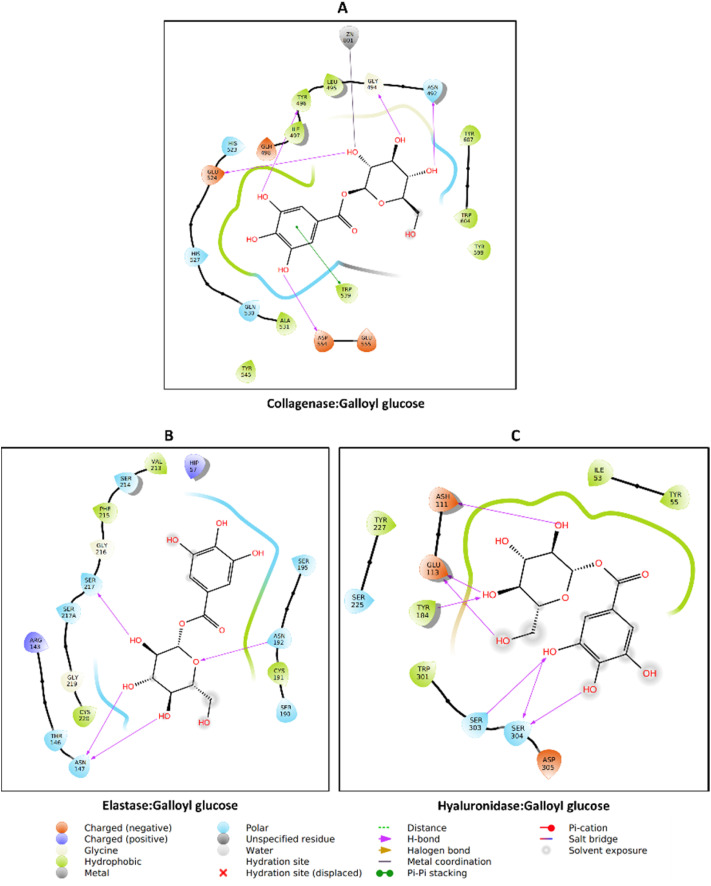
Protein–ligand interaction: (A) collagenase_galloyl glucose, (B) elastase_galloyl glucose, and (C) hyaluronidase_galloyl glucose.

Galloyl glucose binds deeply within the collagenase active-site cleft and is stabilized by an extensive hydrogen-bonding network. The phenolic hydroxyl groups of the galloyl moiety form hydrogen bonds with Asn492, Gly494, Tyr496, Glu524, and Asp554. Aromatic stabilization is provided by Trp539, which participates in π–π interaction with the galloyl ring. Several hydrophobic residues, including Leu495, Ala531, and Trp604 contribute to pocket complementarity and ligand anchoring. Interestingly, the active site zinc metal ion was engaged in metal-acceptor interaction *via* a hydroxyl group (Fig. 11A), suggesting strong inhibition.^116^ This dense interaction pattern supports strong binding and suggests that galloyl glucose may sterically and electrostatically interfere with substrate access to the catalytic region of collagenase.

In elastase, galloyl glucose adopts a more compact binding pose. Hydrogen bonds are primarily formed with Asn147, Asn192, and Ser217 near the catalytic center. Hydrophobic contacts with Val213, Phe215, and Cys220 further stabilize the complex (Fig. 11B). Compared with collagenase, the interaction network is less extensive but more functionally focused, consistent with selective inhibition of elastase.^117^

Galloyl glucose shows the richest interaction profile with hyaluronidase. Multiple hydrogen bonds are observed between ligand hydroxyl groups and Asn111, Glu113, Ser303, and Ser304, reflecting strong electrostatic complementarity within the glycosidase active site. The glucose core engages residues involved in carbohydrate recognition, including Tyr184 through hydrogen bonding, effectively mimicking native hyaluronan contacts.^118^ Additional hydrophobic stabilization is provided by residues such as Ile53 and Tyr55, and Trp301 (Fig. 11C). This combination of sugar-mimetic and polyphenolic interactions explains the broad and stable binding surface observed for hyaluronidase.

## 4 Conclusion

For the first time, we reported the comprehensive chemical profile and biological activities of *E. dodonaei*. The chemical profile revealed that the extracts were rich in flavonoids and gallic acid derivatives. The findings of this study show that *E*. *dodonaei* extracts act far beyond simple antioxidant, enzyme inhibitory, or anti-inflammatory effects. They function as broad-spectrum biological modulators that help maintain cellular redox balance, regulate cytokine responses, and preserve the structural integrity of the extracellular matrix. Our results demonstrate that *E. dodonaei* can establish a coordinated molecular defense in dermal fibroblasts by dampening NF-κB and AP-1 activation, reducing MMP-2/9-driven matrix degradation, limiting intracellular ROS formation, and inhibiting the key enzymes collagenase, elastase, and hyaluronidase. This multi-layered protective mechanism sets the species apart from other members of the genus and offers novel mechanistic evidence supporting its use as a phytotherapeutic candidate for skin repair and the management of inflammation-related or age-associated tissue alterations. In addition, its balanced redox and cytokine-modulating profile suggests potential benefits in broader inflammatory diseases beyond dermatological contexts. Altogether, these insights identify *E. dodonaei* as an overlooked yet promising medicinal plant of the Onagraceae family and encourage its further exploration in evidence-based pharmacological and dermatological research.

## Conflicts of interest

There are no conflicts to declare.

## Supplementary Material

RA-016-D6RA01632D-s001

## Data Availability

Data can be requested from the authors. Supplementary information: the details for phytochemical characterization of extracts. See DOI: https://doi.org/10.1039/d6ra01632d.
