# Supplementary material for: Harnessing the anti-inflammatory, skin-protective, and antioxidant potential of Epilobium dodonaei extracts using in vitro and in silico approaches
Source: RSC Adv. 2026 Apr 8;16(20):18636–56. doi: 10.1039/d6ra01632d (PMC13060018; doi:10.1039/d6ra01632d)
Supplement: RA-016-D6RA01632D-s001 [file RA-016-D6RA01632D-s001.pdf]

Supplementary Information (SI) for RSC Advances.
This journal is © The Royal Society of Chemistry 2026

Phytochemical characterization of extracts

Samples were prepared by mixing approx. 50 mg of extracts with 1 mL of MeOH. Mixtures were sonicated for 10 min and subsequently centrifuged at 13,3000 rpm for 10 min to precipitate suspended material. Analysis was performed by means of UPLC-MS e using a Waters Acquity UPLC coupled to a Xevo G2 ESI-QToF mass spectrometer, operating in negative ion mode (ESI-). Chromatography was performed using a Waters BEH C18 column (2.1 × 50 mm, 1.7 µm) kept at 40 °C as stationary phase, and a mixture of 0.1% formic acid in water (A) and 0.1% formic acid in acetonitrile (B) as the mobile phase. The flow rate was regulated to 300 µL/min. The elution gradient was the following: 0–1 min, 98% A; 11 min, 15% A; 16 min, 0% A; 20 min, 0% A; 21 min, 98% A; 24 min, 98% A. The injection volume was 2 µL. Regarding detection parameters, MS data were acquired in the mass range of 50–2000 Da. The sampling cone voltage was adjusted at 40 V, and the source offset was 80 V. The capillary voltage was set to 3.5 KV. Nitrogen was used as nebulizer gas at a flow rate of 800 L/h. The desolvation temperature was 450 °C. Mass accuracy and reproducibility were maintained by infusing lock mass (leucine–enkephalin, $[M-H]^-$ = 554.2620 m/z) through Lockspray at a flow rate of 20 µL/min. The m/z value of all acquired spectra was automatically corrected during acquisition based on lock mass. MS^e experiment was performed in parallel to collect structural information on the detected metabolites, setting the collision energy to 30 V. Chromatographic and MS data of eluted metabolites were extracted from raw chromatograms and MS spectra using the MarkerLynx XS software (Waters). Tentative identification of metabolites was performed by calculating potential molecular formulas from their isotopic patterns in MS, using the Elemental Composition tool (Waters). The formulas with the highest confidence of fitting (>80%) were then used to search for possible candidates in databases such as PubChem, Chemspider, Natural Products Atlas, and KNApSAck. Identification was confirmed by evaluating the fragmentation patterns in the MS^e spectra.
